# The X-factor in ART: does the use of assisted reproductive technologies influence DNA methylation on the X chromosome?

**DOI:** 10.1186/s40246-023-00484-6

**Published:** 2023-04-21

**Authors:** Julia Romanowska, Haakon E. Nustad, Christian M. Page, William R. P. Denault, Yunsung Lee, Maria C. Magnus, Kristine L. Haftorn, Miriam Gjerdevik, Boris Novakovic, Richard Saffery, Håkon K. Gjessing, Robert Lyle, Per Magnus, Siri E. Håberg, Astanand Jugessur

**Affiliations:** 1 Centre for Fertility and Health, Norwegian Institute of Public Health, Oslo, Norway; 2 Department of Global Public Health and Primary Care, University of Bergen, Bergen, Norway; 3 DeepInsight, 0154 Oslo, Norway; 4 Department of Mathematics, Faculty of Mathematics and Natural Sciences, University of Oslo, Oslo, Norway; 5 Department of Human Genetics, University of Chicago, Chicago, IL USA; 6 Department of Computer Science, Electrical Engineering and Mathematical Sciences, Western Norway University of Applied Sciences, Bergen, Norway; 7 Murdoch Children’s Research Institute, Melbourne, Australia; 8 Department of Paediatrics, University of Melbourne, Melbourne, Australia; 9 Department of Medical Genetics, Oslo University Hospital and University of Oslo, Oslo, Norway

**Keywords:** Epigenetics, Assisted reproductive technology (ART), The Norwegian Mother, Father and Child Cohort Study (MoBa), DNA methylation, Epigenome-wide association study, X chromosome, X-chromosome-wide association study (XWAS), Trio study design, Illumina EPIC array, X chromosome inactivation (XCI)

## Abstract

**Background:**

Assisted reproductive technologies (ART) may perturb DNA methylation (DNAm) in early embryonic development. Although a handful of epigenome-wide association studies of ART have been published, none have investigated CpGs on the X chromosome. To bridge this knowledge gap, we leveraged one of the largest collections of mother–father–newborn trios of ART and non-ART (natural) conceptions to date to investigate sex-specific DNAm differences on the X chromosome. The discovery cohort consisted of 982 ART and 963 non-ART trios from the Norwegian Mother, Father, and Child Cohort Study (MoBa). To verify our results from the MoBa cohort, we used an external cohort of 149 ART and 58 non-ART neonates from the Australian ‘Clinical review of the Health of adults conceived following Assisted Reproductive Technologies’ (CHART) study. The Illumina EPIC array was used to measure DNAm in both datasets. In the MoBa cohort, we performed a set of X-chromosome-wide association studies (‘XWASs’ hereafter) to search for sex-specific DNAm differences between ART and non-ART newborns. We tested several models to investigate the influence of various confounders, including parental DNAm. We also searched for differentially methylated regions (DMRs) and regions of co-methylation flanking the most significant CpGs. Additionally, we ran an analogous model to our main model on the external CHART dataset.

**Results:**

In the MoBa cohort, we found more differentially methylated CpGs and DMRs in girls than boys. Most of the associations persisted after controlling for parental DNAm and other confounders. Many of the significant CpGs and DMRs were in gene-promoter regions, and several of the genes linked to these CpGs are expressed in tissues relevant for both ART and sex (testis, placenta, and fallopian tube). We found no support for parental DNAm-dependent features as an explanation for the observed associations in the newborns. The most significant CpG in the boys-only analysis was in *UBE2DNL*, which is expressed in testes but with unknown function. The most significant CpGs in the girls-only analysis were in *EIF2S3* and *AMOT*. These three loci also displayed differential DNAm in the CHART cohort.

**Conclusions:**

Genes that co-localized with the significant CpGs and DMRs associated with ART are implicated in several key biological processes (e.g., neurodevelopment) and disorders (e.g., intellectual disability and autism). These connections are particularly compelling in light of previous findings indicating that neurodevelopmental outcomes differ in ART-conceived children compared to those naturally conceived.

## Background

The use of assisted reproductive technologies (ART) has been on the rise in most parts of the world since the first baby was born to *in vitro* fertilization (IVF) in 1978 [1, 2]. The trend of declining fecundity and greater reliance on ART to conceive is expected to persist in the future, as egg-freezing gains more acceptance in contemporary societies and more couples choose to postpone childbearing [3–5]. As the clinical and laboratory procedures for ART coincide with the developmental window in which the early embryo undergoes extensive epigenetic remodeling [6–8], it is critical to determine whether the ART procedures themselves or some underlying mechanisms related to parental characteristics (e.g., parental infertility) are responsible for the observed epigenetic differences between ART and non-ART newborns. A number of epigenome-wide association studies (EWASs) of ART have been published in recent years [9–17] and have already contributed substantially to our current understanding of epigenetic changes associated with ART. However, none of these studies have investigated the effect of epigenetic markers on the X chromosome.

Until recently, most genome-wide association studies (GWASs) were also performed almost exclusively on autosomes, leaving out single-nucleotide polymorphisms (SNPs) on the X chromosome, even though this chromosome constitutes $\sim$5% of the human genome and houses $\sim$1000 genes, several of which have been associated with complex traits [18, 19]. The main reason for this exclusion is that the initial methods for GWAS were primarily designed for autosomal markers, as analyzing different X chromosome contents in males and females comes with its own set of analytic challenges [20]. To fill this knowledge gap, we and others have developed a suite of biostatistical tools for analyzing X-linked SNPs both individually and as haplotypes [21–31]. Currently, there is a similar trend of systematic exclusion of CpGs on the sex chromosomes in the vast majority of EWASs, which may result in overlooking important associations.

There are several reasons why X chromosome markers are less tractable to analyze than autosomal markers. First, one needs to account for X chromosome inactivation (XCI) in which one of the X chromosomes in female somatic cells is randomly selected and transcriptionally inactivated in early embryonic development [32, 33]. This crucial mechanism ensures a balanced dosage of X-linked genes in males and females [34–36]. However, XCI is not complete in humans, with approximately 12% of the genes reported to escape XCI and a further 15% differing in their XCI status across individuals, tissues, and cells [33, 37–39]. Second, the analysis of X-linked markers is complicated by genes in the pseudoautosomal regions (PARs), which are expressed in a similar fashion to autosomal genes as a consequence of escaping XCI [40, 41]. Third, the gradual loss of the X chromosome with age [42] may further complicate the analysis of X-linked markers when comparing cohorts that differ significantly with age.

Despite these challenges, taking X chromosome markers into account in a GWAS or EWAS is important based on the following observations: (a) genes on the X chromosome are known to play essential roles in transcriptional regulation of autosomal genes [43, 44], (b) several traits show a consistently higher prevalence in one sex, and (c) there are distinct physical differences between the sexes (sexual dimorphism) [45]. All of these features might stem from sex-specific differences which may be especially relevant for differences occurring *prior* to gonadal differentiation, i.e., differences that are solely attributable to sex chromosome content rather than those induced by gonadal and hormonal changes [34, 35, 46]. Although a wide variety of traits are known to exhibit sex-specific DNA methylation (DNAm) signatures on the autosomes [47–57], less is known about the presence of such signatures on the X chromosome, possibly due to the overall lack of focus on X-linked markers and the dearth of X-chromosome-wide association studies (XWASs) conducted to date. The few XWASs published thus far include an investigation of CpGs influenced by cigarette smoking, an exploration of differential chronological aging in males versus females, and a study of DNAm changes associated with aging on the X and Y chromosomes [58–61].

Given these important knowledge gaps, our main objective was to examine sex-specific differences in DNAm profiles on the X chromosome by contrasting ART and naturally conceived newborns. We used one of the largest case–control collection of mother–father–newborn trios of ART and non-ART conceptions to date [62], stemming from the Norwegian Mother, Father, and Child Cohort Study (MoBa) [63]. The analyses were stratified by sex and adjusted for potential confounding factors (mother’s age, smoking status, BMI, and primiparity, as well as parental DNAm at each CpG). As an external check of our results from the MoBa cohort, we analyzed data from the Australian ‘Clinical review of the Health of 22–33 years old conceived with and without ART’ (CHART) cohort [64, 65], which was the only available external ART cohort to which we could compare our MoBa results.

## Results

In the discovery cohort (MoBa), we analyzed DNAm data from 982 ART and 963 non-ART mother–father–newborn trios. These data were generated on the Illumina EPIC platform using DNA extracted from peripheral blood in adults and cord blood in newborns (for details, see Methods and ref. [62]). Our main aim was to identify differences in DNAm in ART versus non-ART newborns, both at a single-CpG level across the entire X chromosome (XWAS) and at a regional level where we searched for differentially methylated regions (DMRs). All the analyses were performed separately for boys and girls.

In the main model, we adjusted for known confounders (mother’s age, smoking status, BMI, and primiparity). In addition, we tested three adjusted models, where we included: *(i)* parental DNAm at each CpG, *(ii)* birthweight and gestational age of the newborn, and *(iii)* all the covariates from *(i)* and *(ii)*. All the analyses were stratified by sex. We also explored co-methylation patterns between significant CpGs identified by the above analyses as well as other CpGs in the immediate flanking regions.

**Fig. 1:**
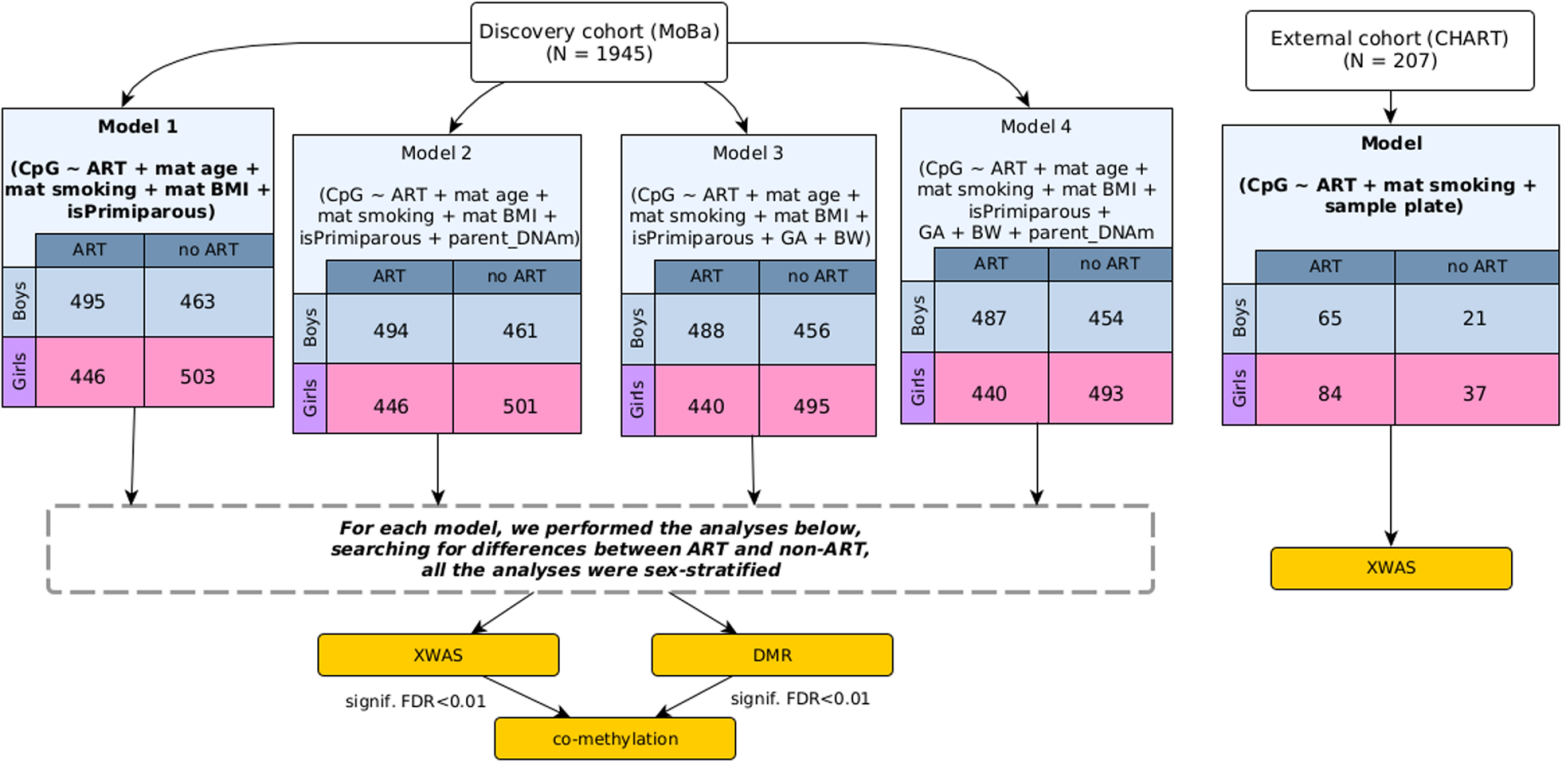
Overview of the analytic pipeline. We refer to **Model 1** as the ‘main model’ throughout this paper and focus primarily on significant findings from this model. The other models are referred to as ‘adjusted models’ and treated as sensitivity analyses. Abbreviations used in the figure: CpG = cytosine-phosphate-guanine; ART = assisted reproductive technologies; mat = maternal; BMI = body mass index; BW = birthweight; GA = gestational age; XWAS = X-chromosome-wide association study; DMR = differentially methylated region; FDR = false discovery rate; MoBa = The Mother, Father, and Child Cohort Study; and CHART = The ‘Clinical review of the Health of adults conceived following Assisted Reproductive Technologies’ study

In the external cohort (CHART), we analyzed DNAm data, also generated on the EPIC array, from 149 ART-conceived and 58 non-ART newborns. These analyses are outlined in Fig. 1 and detailed in the Methods section.

### Differences between ART and non-ART newborns and their parents in the MoBa cohort

The ART parents were older than the non-ART parents, and the ART newborns weighed less than the non-ART newborns (Table 1). Fewer of the ART mothers smoked during pregnancy than the non-ART mothers, but, intriguingly, a higher proportion of the ART mothers were *past* smokers.

**Table 1:** Characteristics of the discovery cohort (MoBa)

Characteristic	Non-ART ($N = 983$)$^1$	ART ($N = 962$)$^1$	*p* value$^2$
Male (%)	470 (48%)	505 (52%)	0.039
Maternal age (years)	30 (27–33)	33 (31–36)	<0.001
Paternal age (years)	32 (29–36)	35 (32–38)	<0.001
Gestational age (weeks)	40 (39–41)	40 (39–41)	0.090
*(No data)*	4	0	
Birth weight (g)	3650 (3330–3970)	3540 (3190–3850)	<0.001
*(No data)*	0	1	
First child	461 (47%)	673 (70%)	<0.001
Maternal BMI (kg/m$^2$)	23 (21–26)	23 (21–26)	0.4
*(No data)*	14	17	
Maternal smoking			<0.001
*Never*	490 (50%)	494 (52%)	
*Past smoker*	253 (26%)	358 (37%)	
*1st trimester*	132 (13%)	62 (6.5%)	
*1st trimester and after*	104 (11%)	44 (4.6%)	
*(No data)*	4	4	

$$^1$$ n (%); median (IQR)

$^2$ Pearson’s chi-squared test; Wilcoxon rank-sum test

**Fig. 2:**
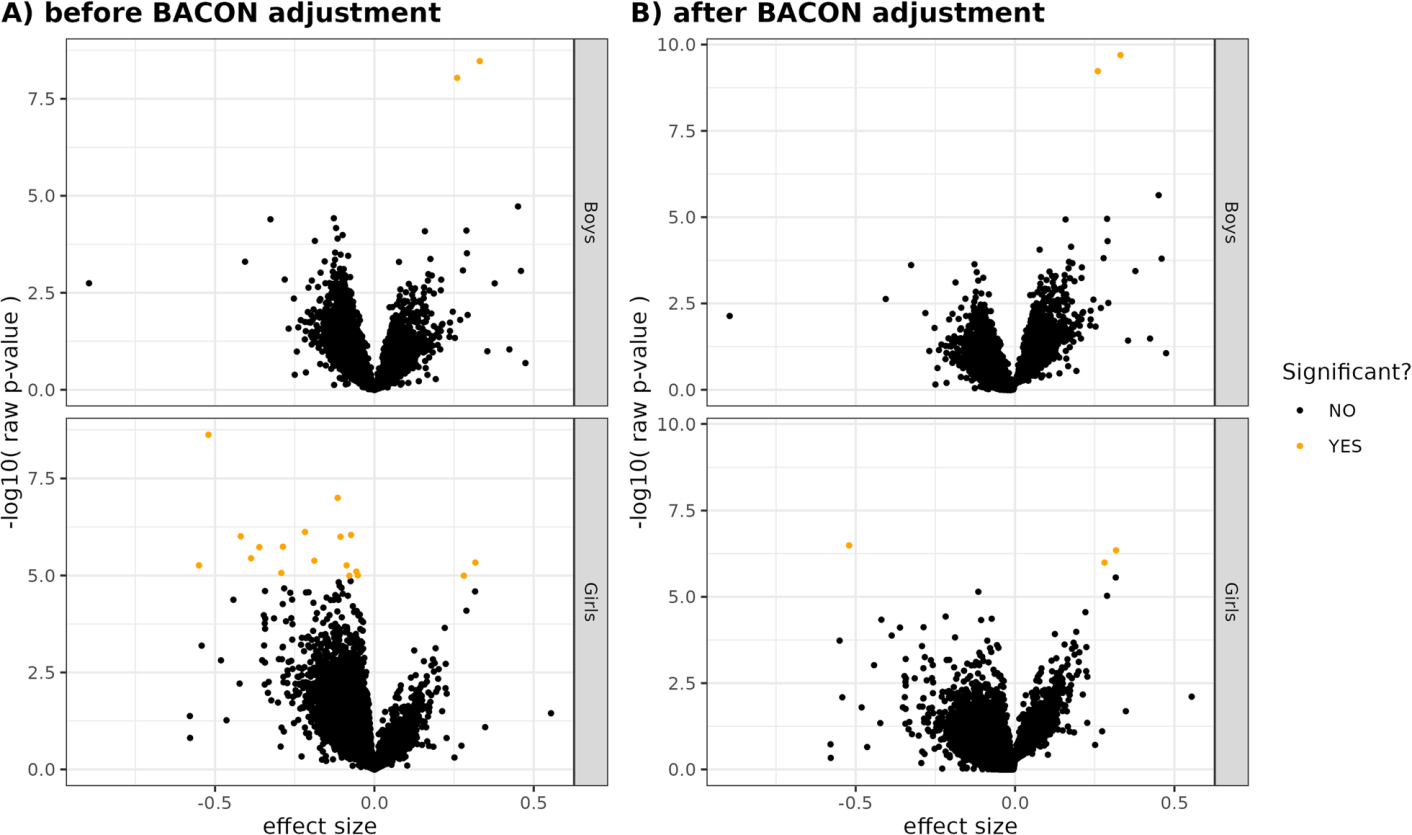
Effect sizes versus $-\log _{10} p$ values for each of the X-linked CpGs included in the analysis of the MoBa cohort. Significant findings at FDR < 0.01 are highlighted in orange. The data are presented before (**A**) and after (**B**) *p* value adjustment with the BACON algorithm. This figure shows the results of the analysis when applying the main model, while all the models are included in Additional file 1: Figure S3.

Figure 2 highlights the general trends in the XWAS results for boys and girls separately, before and after controlling for inflation using the R package BACON (see Methods for details). Effect sizes for the CpGs were dominated by global hypomethylation (overall lower DNAm level in the ART newborns). Figure 2 also illustrates the efficacy of BACON in reducing inflation in the *p* values. A similar figure showing the results of all the models tested can be found in Additional file 1: Figure S3.

### Several CpGs were significantly differentially methylated between ART and non-ART in the MoBa cohort

**Fig. 3:**
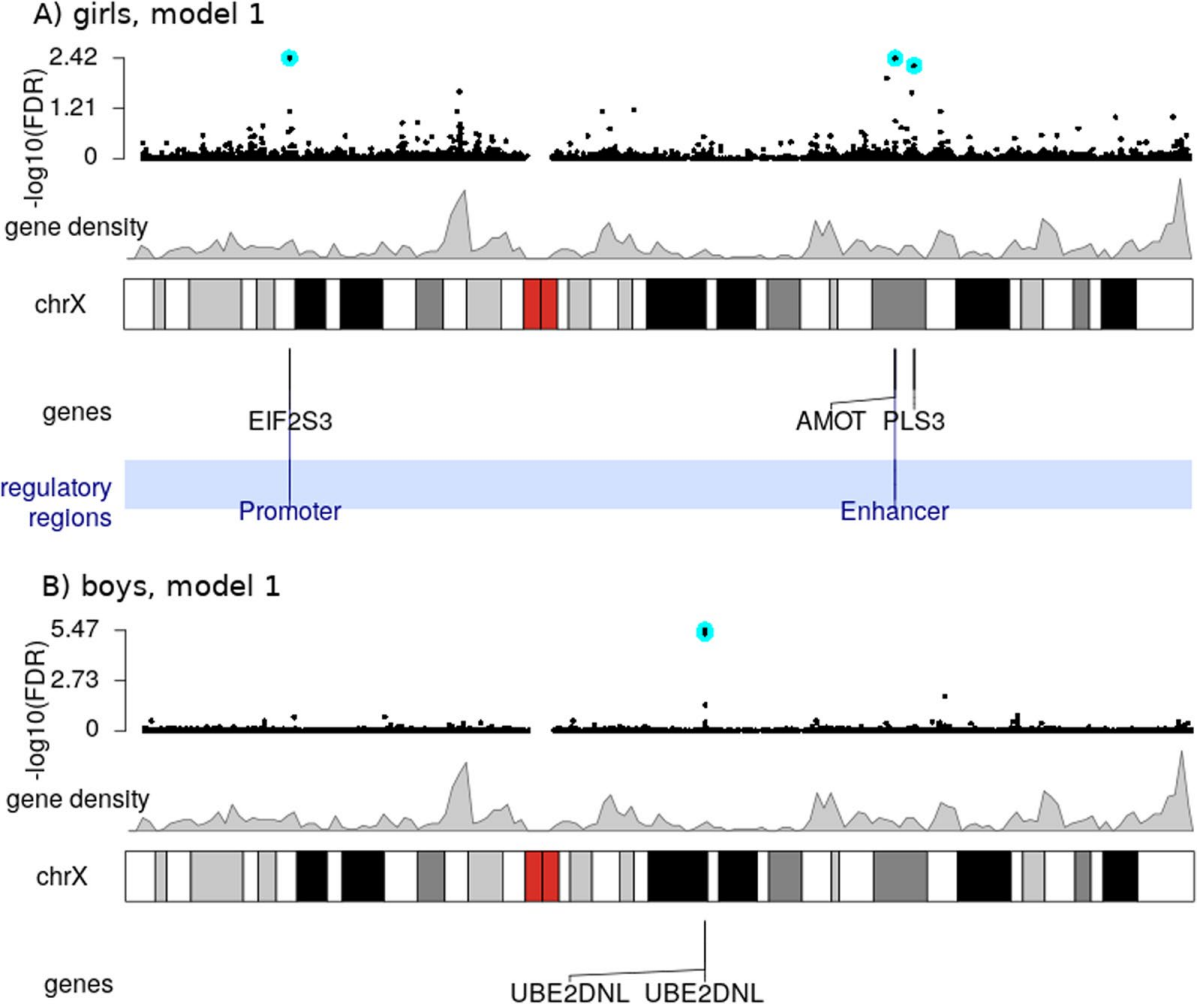
Results of the XWAS of girls (**A**) and boys (**B**) based on the main model (**Model 1**; CpG $\sim$ ART + maternal age + maternal smoking + maternal BMI + primiparity). The top plot in each panel is a Manhattan plot of all the tested CpGs. The genomic locations of the most significant findings (FDR < 0.01) are highlighted by cyan-colored circles. Immediately beneath is a line plot of gene density, the chromosomal bands, and any genes and/or regulatory regions that overlap with the significant findings

We identified three significantly differentially methylated CpGs in the girls-only analysis (cg25034591, cg13866977, and cg26175661) and two CpGs in the boys-only analysis (cg00920314, cg04516011), all at a false discovery rate-adjusted (FDR-adjusted) *p* value < 0.01. Strikingly, there was no overlap in the location of the significant findings between the girls-only and boys-only analyses (see Fig. 3 for the location of findings from the main model and Additional file 2 for the significant results for all four statistical models). Additionally, tables with all the results are available in GitHub, at https://github.com/folkehelseinstituttet/X-factor-ART.

Adjusting for parental DNAm in the MoBa sample enabled ruling out parental characteristics as the reason for the observed DNAm differences. When the results of the main model (Model 1 of Fig. 1) were contrasted with those of the adjusted models (Models 2–4 of Fig. 1), there was no significant change in the findings in the boys-only analyses (Additional file 2). By contrast, only cg25034591 and cg13866977 remained significant across all models in the girls-only analyses. These results suggest that the differential methylation at these sites is more likely the result of the ART procedures themselves rather than parental DNAm.

We performed bootstrap analyses to evaluate the consistency with which the significant CpGs were retained (see Methods for details). The two significant CpGs in the boys-only analyses (cg00920314 and cg04516011) showed a high degree of consistency. They were significant 54% and 47% of the time, respectively, which is substantially higher than the next CpG on the ranked list (cg00243584 at 9%). In the girls-only analyses, cg25034591 was significant in 51% of the bootstrap samples, but the other two CpGs were not as consistent (cg13866977 at 25% and cg26175661 at 19%, occupying positions six and 14 on the ranked list, respectively). The full list of CpGs found to be significant at least once, and the proportion of times a given CpG was found to be significant, are provided in Additional file 4 for the boys-only analyses and in Additional file 5 for the girls-only analyses. These tables are also provided in the GitHub repository.

The two significant CpGs detected in the boys-only analysis are adjacent and located within the gene ‘Ubiquitin conjugating enzyme E2 D N-terminal like’ (*UBE2DNL*) (Fig. 3). In contrast, the significant CpGs in the girls-only analysis are located in different chromosomal regions, i.e., within ‘Eukaryotic translation initiation factor 2 subunit gamma’ (*EIF2S3*), ‘Angiomotin’ (*AMOT*), and ‘Plastin 3’ (*PLS3*) (Fig. 3). Two of the CpGs are located within promoter regions and one within an enhancer. See Table 2 for a summary of the genes.

**Table 2:** Summary of the genes and loci identified in the current XWAS

Sex	Gene name (location; ensembl ID)	Full gene name (MIM entry)	Gene function$^1$	Refs
Girls	*EIF2S3* (Xp22.11; ENSG00000130741)	Eukaryotic translation initiation factor 2 subunit gamma (MIM:300161)	This gene encodes the core subunit of eukaryotic translation initiation factor 2 (eIF2). This gamma subunit is the largest component of a heterotrimeric GTP-binding protein which is essential for protein synthesis. Hemizygous mutations in *EIF2S3* cause an X-linked syndrome called ‘mental retardation, epileptic seizures, hypogonadism and -genitalism, microcephaly, and obesity’ (MEHMO). *EIF2S3* has also been reported to escape XCI.	[33, 76, 77, 143, 144]
Girls	*AMOT* (Xq23; ENSG00000126016)	Angiomotin (MIM:300410)	This gene belongs to the motin family of angiostatin-binding proteins containing conserved coiled-coil domains and C-terminal PDZ binding motifs. *AMOT* is predominantly expressed in endothelial cells of capillaries and in larger vessels of the placenta where it may mediate the inhibitory effect of angiostatin on tube formation and the migration of endothelial cells toward growth factors during the formation of new blood vessels.	[145–147]
Girls	*PLS3* (Xq23; ENSG00000102024)	Plastin 3 (MIM:300131)	Plastins comprise a family of actin-binding proteins that are conserved throughout eukaryote evolution. They are expressed in most tissues of higher eukaryotes. Two ubiquitous plastin isoforms (L and T) have been identified in humans.	
Boys	*UBE2DNL* (Xq21.1; ENSG00000229547)	Ubiquitin conjugating enzyme E2 D N-terminal like (pseudogene)	*UBE2DNL* is labeled ‘pseudogene’ in various gene databases, but it is reported to be expressed in testis.	Not applicable

$^1$ Information on gene function was collated from various sources, including NCBI’s Entrez Gene (https://www.ncbi.nlm.nih.gov/gene), Gene Cards (https://www.genecards.org/), the Online Inheritance in Man (OMIM) (https://omim.org/), and the cited references in the last column

### Patterns of co-methylation around the significant CpGs in the MoBa cohort

**Fig. 4:**
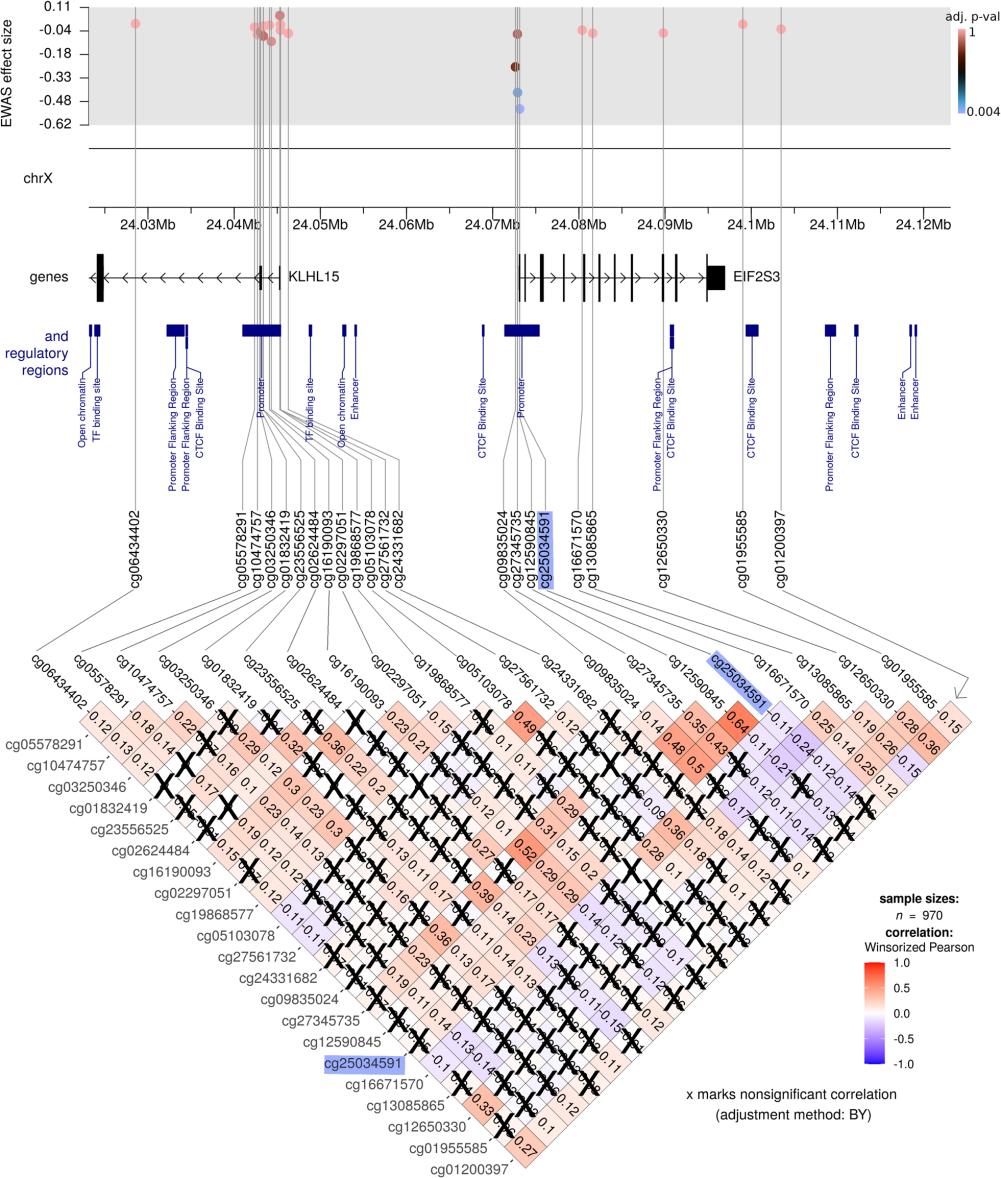
Co-methylation patterns in the region flanking the most significant XWAS finding (cg25034591; highlighted in blue) in the girls-only analysis. The top part of the figure shows the effect sizes and FDR-adjusted *p* values for the CpGs within 50 kb of cg25034591. The X chromosome coordinates are provided directly underneath. The middle part of the plot shows the location of genes and regulatory regions based on GRCh37 annotations. The bottom part of the figure shows the DNAm correlation matrix, where the color gradient indicates the strength and direction of correlation of DNAm level for each pair of CpGs. Note that the correlation coefficients are provided inside each matrix element (each diamond), and nonsignificant correlations are crossed out (significance was estimated based on adjusted p-values, using the Benjamini-Yakuteli method)

**Fig. 5:**
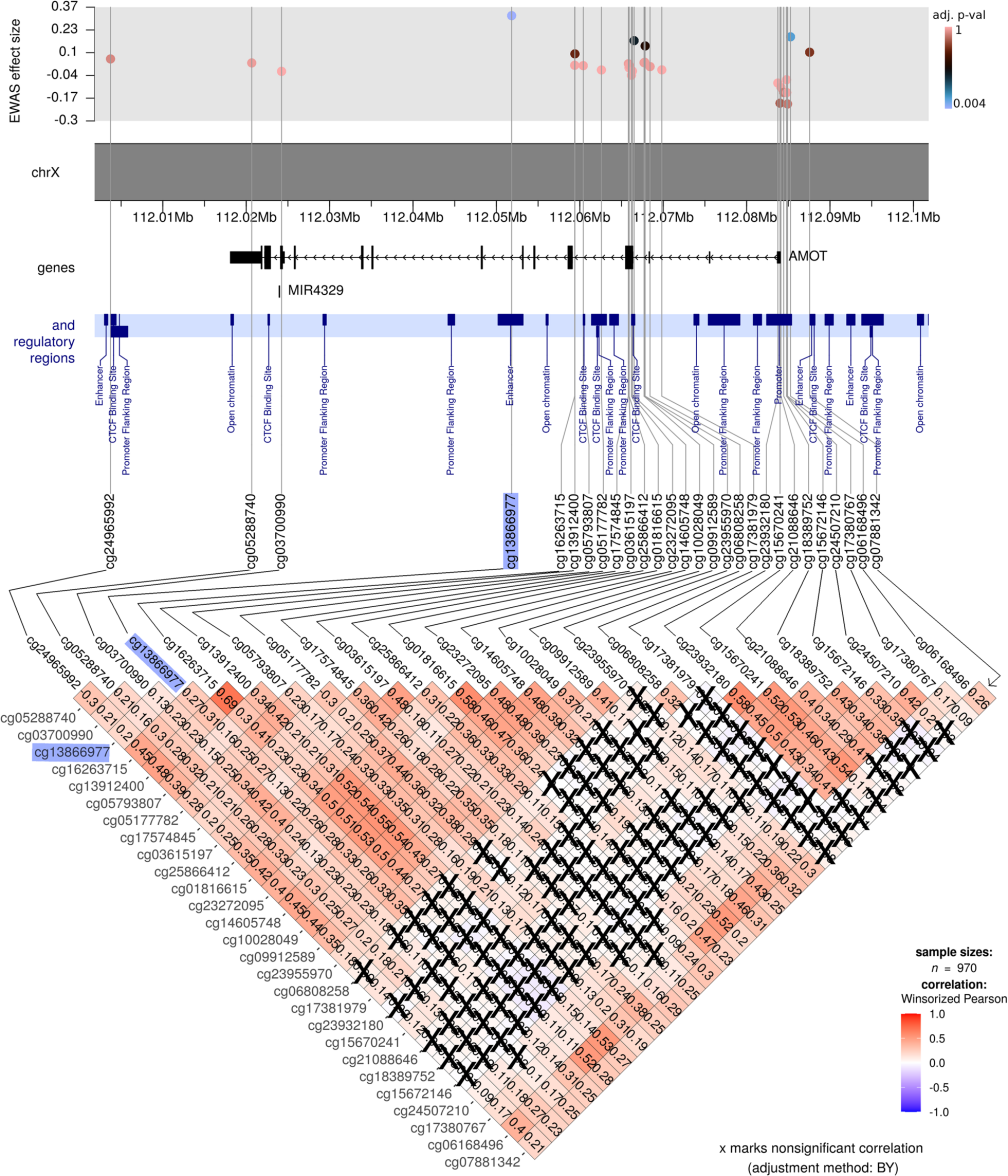
Co-methylation patterns among CpGs within 50 kb of cg13866977 (highlighted in blue). The rest of the figure legend is similar to that of Fig. 4 and will therefore not be repeated here or in the remaining co-methylation figures below

**Fig. 6:**
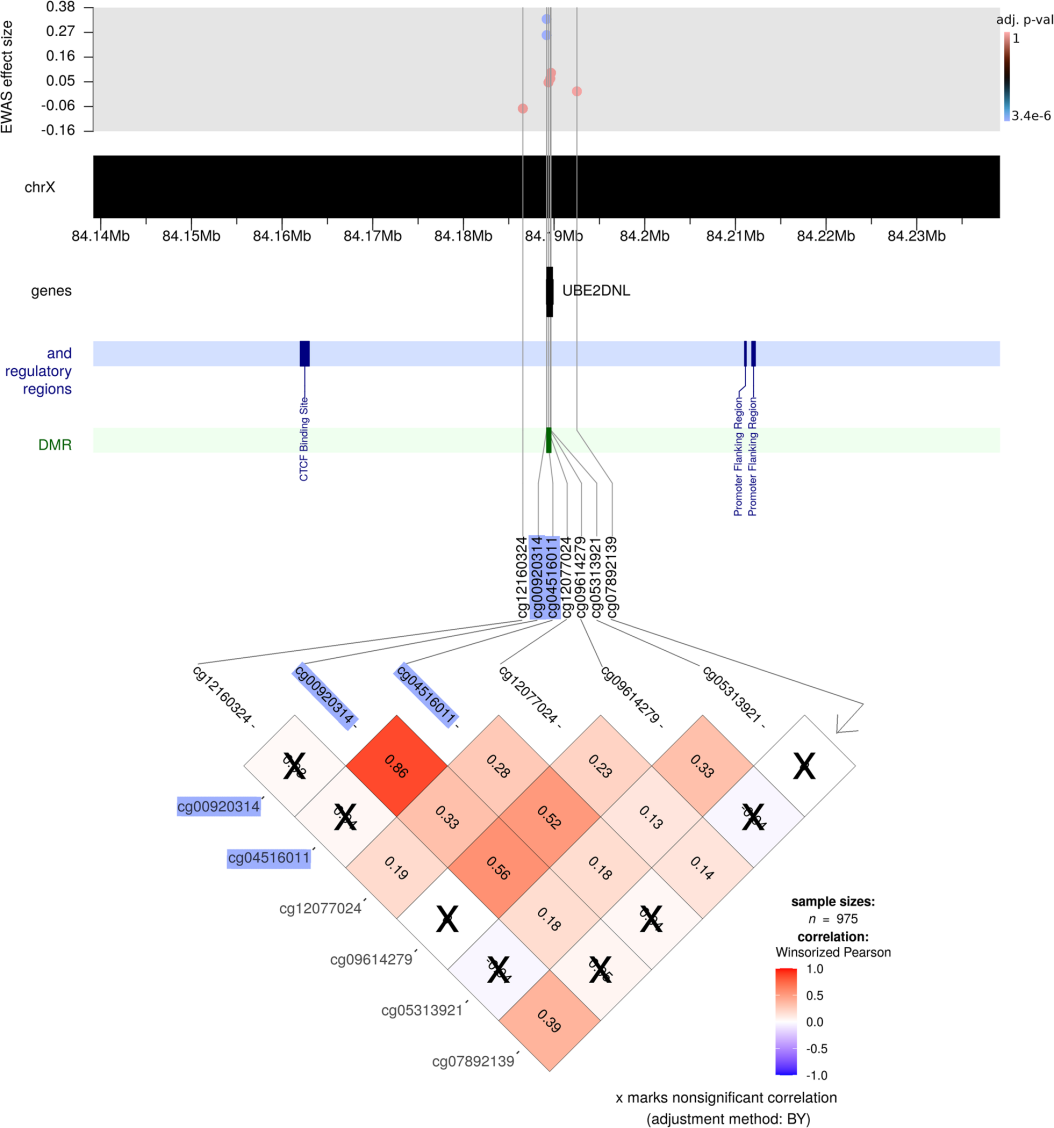
Co-methylation patterns in the area flanking the most significant XWAS finding in the boys-only analysis (cg00920314; highlighted in blue). As with the co-methylation figures for the girls-only analyses, the top part of the plot shows the effect sizes and *p* values for the CpGs within 50 kb of cg00920314, which also includes the next most significant CpG, cg04516011 (also highlighted in blue). In addition, the dark-green bar in the plot indicates a DMR in this region

**Fig. 7:**
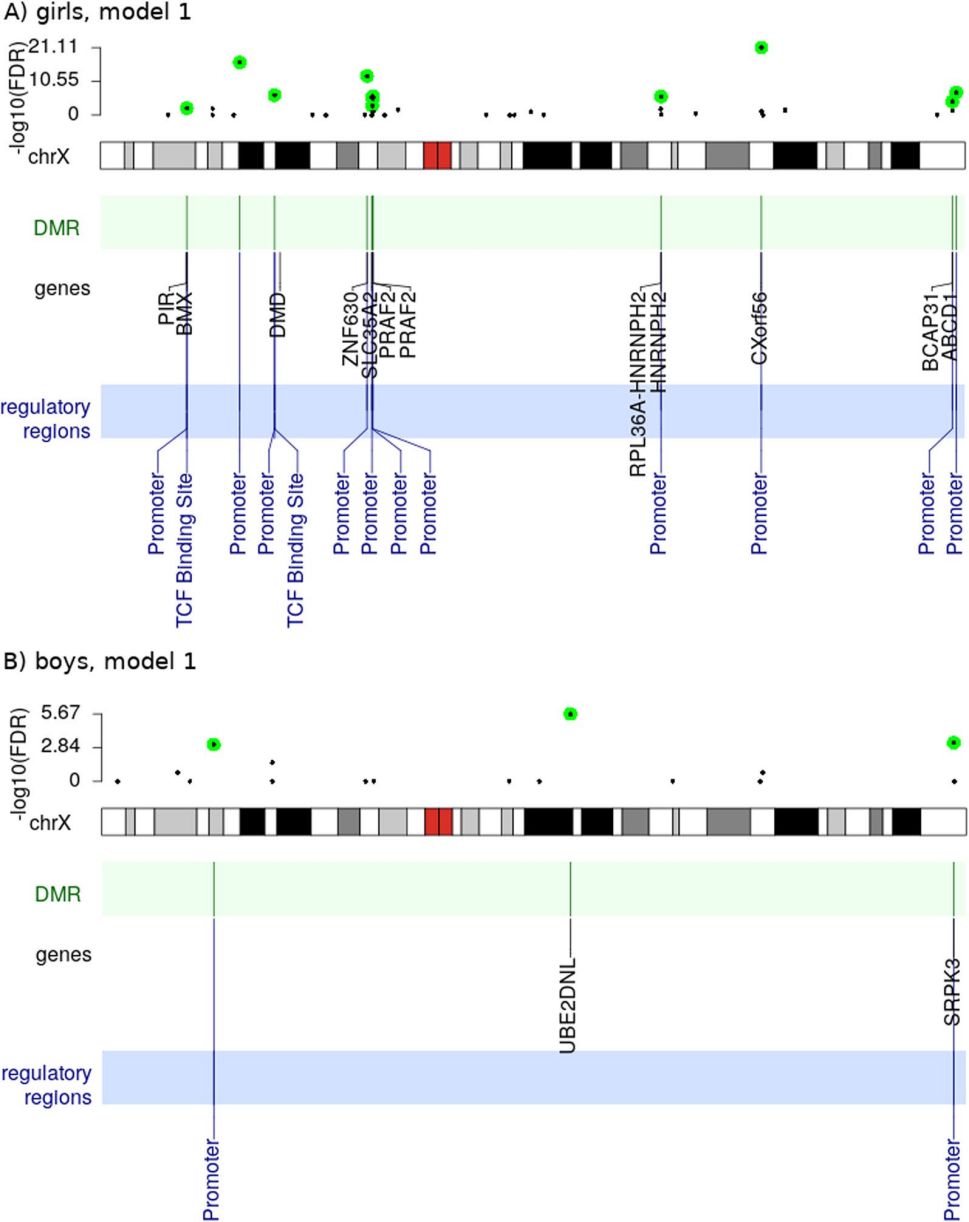
Location of DMRs on the X chromosome. The girls-only analysis is shown in panel A and the boys-only analysis in panel B. Note that we only show the results of the main model (**Model 1**). The top part of each panel shows *p* values for all the DMRs that contain at least three CpGs. The FDR-adjusted *p* values < 0.01 are marked in green. The bottom part of each panel marks genes and regulatory regions harbored by the significant DMRs

Analyzing clusters of DNAm can be more informative than scrutinizing one CpG at a time, as it may, for example, help in identifying co-methylation patterns and regions that may be important from a population-epigenetic perspective [66]. Accordingly, we examined regions of 50 kb around each significant CpG detected in our XWAS (Figures 4–6 show important examples). This led to the identification of clusters of positively correlated CpGs often mapping to a promoter region (see Fig. 4). We also observed clusters of CpGs within gene body regions, such as cg13866977 which was positively correlated with 16 other CpGs across the *AMOT* region (Fig. 5). Overall, the direction of correlation patterns in the promoter region and gene body was opposite to each other, which is as expected and consistent with gene expression patterns that are typical for these regions [67, 68]. To illustrate, a cluster of three CpGs in the promoter region near *EIF2S3*, with positive correlation between the CpGs, was also positively correlated with cg25034591 (Fig. 4). In contrast, a different cluster of four CpGs within the *EIF2S3* gene body, which were positively correlated with one another, was *negatively* correlated with cg25034591. The co-methylation analysis of the significant findings in the boys-only XWAS (Fig. 6) indicated that both significant CpGs are located within a cluster of correlated CpGs and are also part of a DMR located at chr X:84,189,179-84,189,658 (GRCh37) that harbors four CpGs (see the section below and Fig. 7).

### DMR analysis in ART and non-ART newborns in the MoBa cohort

**Fig. 8:**
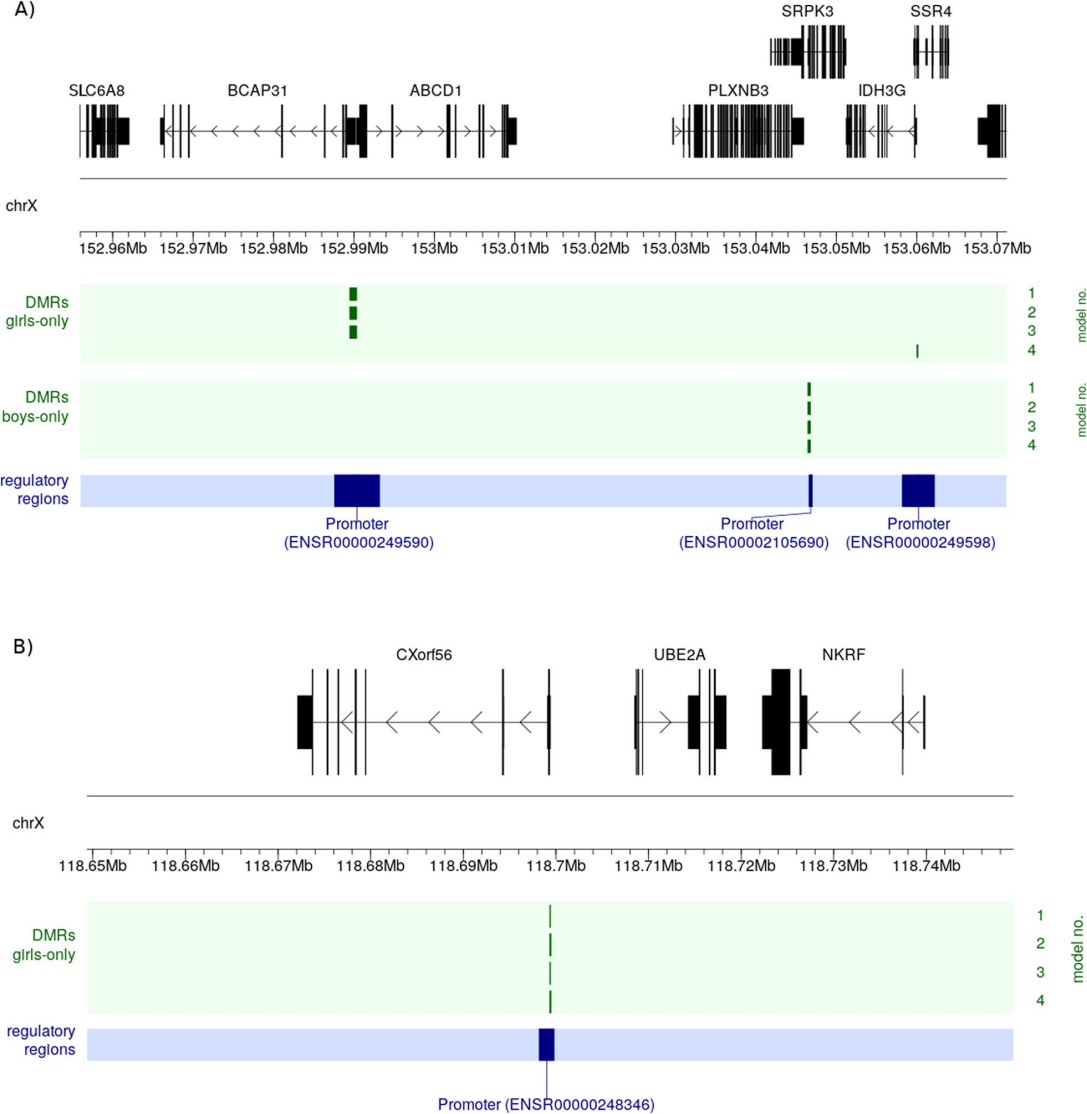
Zooming in on genomic features around selected significant DMRs. **A** These significant DMRs detected in the girls-only and boys-only analyses where the only ones localized in the vicinity of each other; the DMRs’ positions are marked by green vertical bars in four lines, where each line corresponds to a specific statistical model (**Models 1–4**; right-hand side). **B** The most significant DMR detected in the girls-only analyses

We identified 12 significant DMRs in the girls-only analysis and three in the boys-only analysis (main model, Fig. 7 and Additional file 3). We considered a DMR as being statistically significant if it contained three or more CpGs and had an FDR-adjusted *p* value < 0.01. The number of significant DMRs varied only slightly between the main and the adjusted model (see Additional file 1: Figures S4–S6 and the table with all significant DMR findings in Additional file 3); notably, we found eight DMRs in the girls-only analysis and two in the boys-only analysis that were shared across all the models tested. The majority of these DMRs were located in promoter regions. See Additional file 3 for more details as well as the GitHub repository for all the results. In one instance, a DMR included a CpG that was significantly associated with the ART phenotype in boys (Fig. 6). Overall, however, there was only one instance where the DMRs in boys and girls were near each other (Fig. 8, panel A).

### Analyses in an independent cohort—CHART

**Fig. 9:**
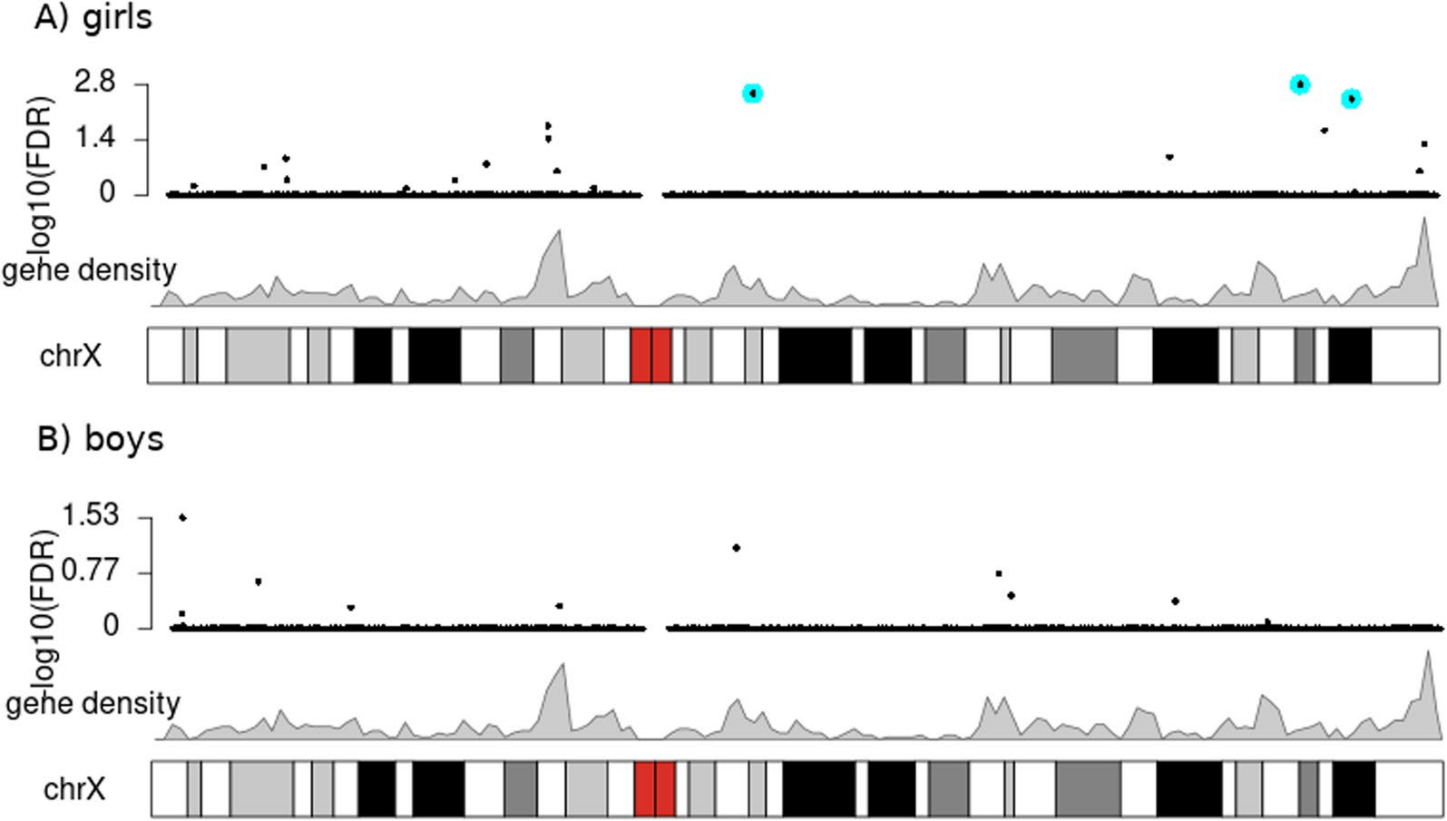
Results of the XWAS of girls (**A**) and boys (**B**) from CHART cohort, based on an analogous model to the main model (CpG $\sim$ ART + maternal smoking). The top plot in each panel is a Manhattan plot of all the tested CpGs. The genomic locations of the most significant findings (FDR < 0.01) are highlighted as cyan-colored circles. Immediately beneath is a line plot of gene density and chromosomal bands

To check our results from the MoBa cohort, we ran an analogous model to our main model on an independent dataset from the Australian CHART study (https://lifecourse.melbournechildrens.com/cohorts/art/, see Fig. 1 and Methods). Due to the substantially smaller sample size of the CHART cohort (149 ART and 58 non-ART newborns), none of the associations were statistically significant (FDR < 0.01) in boys and there were only three significant associations in girls (cg26018312, cg25174364, and cg15133558; Fig. 9). While these CpGs are located in different areas than the significant results from the MoBa dataset, they were not linked to any gene or regulatory region, and we were thus unable to analyze these further. However, the results did point to changes in DNAm associated with ART status at CpGs in *EIF2S3* and *AMOT* for girls (Additional file 1: Figure S7). Here, the CpGs differed from those in the MoBa cohort because of differences in the quality of DNAm readings in the two cohorts. We show the CpGs within the *AMOT* gene that were also available in the MoBa dataset in Additional file 1: Figure S8. Moreover, there was a 401 bp-long DMR in *UBE2DNL* in the boys-only analyses, which contained four hypomethylated probes (data not shown). Finally, we observed differences in DNAm levels at cg04516011 and cg00920314 in boys (Additional file 1: Figure S9), which were also identified in the larger MoBa sample (see Additional file 1: Figure S10 for a comparison). All the results, including *p* values and effect sizes, are available online (GitHub repository at https://github.com/folkehelseinstituttet/X-factor-ART).

## Discussion

We investigated sex-specific differences in DNAm levels on the X chromosome between newborns conceived through the use of ART and those conceived naturally. Equipped with the largest collection of ART trios to date, we searched for DNAm differences at single-CpG sites as well as in regions. Specifically, we ran four separate models to check for the effect of various potential confounders, including parental DNAm. We also performed an external check of the results from the MoBa cohort using the main model on a smaller, independent cohort of ART newborns from Australia (CHART).

### Characteristics of the ART and non-ART participants in the MoBa cohort.

The ART parents in our study were older than the non-ART parents, and the ART newborns weighed less than the non-ART newborns. Both of these observations are consistent with previous findings [69–73]. Furthermore, fewer of the ART mothers smoked during pregnancy than the non-ART mothers, but, intriguingly, a higher proportion of the ART mothers were past smokers. The observation that more ART mothers were past smokers is noteworthy in light of previous findings linking smoking to impaired fertility in both men and women [74]. Furthermore, a meta-analysis of 21 studies [75] revealed significant associations between smoking at the time of ART treatment and lower success rate for a number of clinical outcomes of ART. Specifically, smoking was associated with lower odds of live birth per cycle, lower odds of clinical pregnancy per cycle, higher odds of spontaneous miscarriage, and higher odds of ectopic pregnancy [75].

### Significant sex-specific DNAm differences in ART and non-ART newborns

The results of our XWASs of the MoBa data showed significant sex-specific DNAm differences in ART and non-ART newborns. These differences remained significant even after adjusting for several confounders known to be associated with cord-blood DNAm. The results also revealed more differentially methylated CpGs and DMRs in girls than boys, with a slightly lower overall X-chromosome-wide methylation. The differentially methylated CpGs in our study were mostly located in promoters controlling genes involved in several key developmental processes (e.g., neurodevelopment) and disorders (e.g., intellectual disability and autism).

### Differential DNAm at cg25034591 suggests upregulation of several genes involved in transcription and translation processes

In the MoBa cohort, the most significant CpG associated with ART in the girls-only analyses, cg25034591, is located in *EIF2S3* and a promoter region (ensembl ID: ENSR00000245352). This promoter region regulates ten genes (https://www.genecards.org/Search/Keyword?queryString=ENSR00000245352, see Additional file 1: Table S1), five of which encode a highly interconnected group of proteins with important functions in the regulation of transcription and translation (https://version-11-5.string-db.org/cgi/network?networkId=b4AYVO1yPaIh). The DNAm patterns around cg25034591 form two distinct clusters, one containing a set of positively correlated downstream CpGs and the other a set of negatively correlated upstream CpGs (Fig. 4). This pattern indicates that cg25034591 does not act alone, but operates in concert with other neighboring CpGs. This result was also supported by the independent data from the CHART cohort, where two other CpGs in *EIF2S3* displayed marked differences in DNAm in ART versus non-ART girls (Additional file 1: Figure S7). These CpGs were not present in the MoBa sample analyses because they had been excluded after quality control.

Mutations in *EIF2S3* cause MEHMO, a rare X-linked syndrome characterized by intellectual disability, epilepsy, hypogonadism, hypogenitalism, microcephaly, and obesity [76–78]. Interestingly, both *EIF2S3* and cg25034591 have been reported to escape XCI [33, 59]. We also find evidence for this in our data; notably, the $\beta$-values for DNAm at cg25034591 were within the range 0.00009$-$0.018 in ART-conceived girls and within 0.00016$-$0.032 in those naturally conceived. It is thus plausible that ART interferes with the escape of XCI at this CpG, leading to an upregulation of genes controlled by the promoter ENSR00000245352. Interestingly, one study reported that impaired imprinted X-chromosome inactivation was responsible for the skewed sex ratio observed after in vitro fertilization [79]. It would therefore be worthwhile to examine XCI in more detail in future analyses.

### Interpreting the relevance of the findings in the context of ART

The second most significant CpG in girls, cg13866977, lies within a regulatory region and an intron of *AMOT*. This CpG was originally annotated to a region defined as an ‘enhancer’ in the GRCh37 (hg19) version of the genome, but was subsequently changed to ‘promoter flanking region’ in the newer GRCh38 (hg38) genome build (ensembl regulatory ID: ENSR00000912938). It is not unusual for the definition and location of an annotation to change from one genome version to another, especially when the distinction between a promoter and an enhancer becomes blurred as a result of sharing several properties and functions [80]. A perhaps more suitable annotation for cg13866977 in this case would have been ‘transcription regulatory element.’ Furthermore, GeneHancer [81] lists this regulatory region as a putative enhancer (https://www.genecards.org/Search/Keyword?queryString=ENSR00000912938) for four genes, one of which is *AMOT* (Additional File 1: Table S1). *AMOT* is a member of the motin family of angiostatin-binding proteins. This gene is especially relevant for ART since it is expressed in placental vessels and the endothelial cells of capillaries, with reported links to premature births [82]. Nevertheless, interpreting the relevance of this finding in the context of ART is not straightforward.

The above-mentioned promoter, ENSR00000912938, is particularly active in six different types of tissues, including the placenta. However, there is no evidence of its activity in cord blood. These observations are based on the ensembl visualization of experimental data showing various histone marker states and DNase1 activity for this promoter (http://www.ensembl.org/Homo_sapiens/Regulation/Summary?db=core;fdb=funcgen;r=X:112806973-112809972;rf=ENSR00000912938). Furthermore, according to the Genotype-Tissue Expression (GTEx) database [83], neither *AMOT* nor ‘LHFPL tetraspan subfamily member 1’ (*LHFPL1*), another protein-coding gene controlled by this regulatory region, is transcribed in blood, which is paradoxical given that the DNAm data in both the MoBa and the CHART cohort were generated from newborn’s cord blood.

**Fig. 10:**
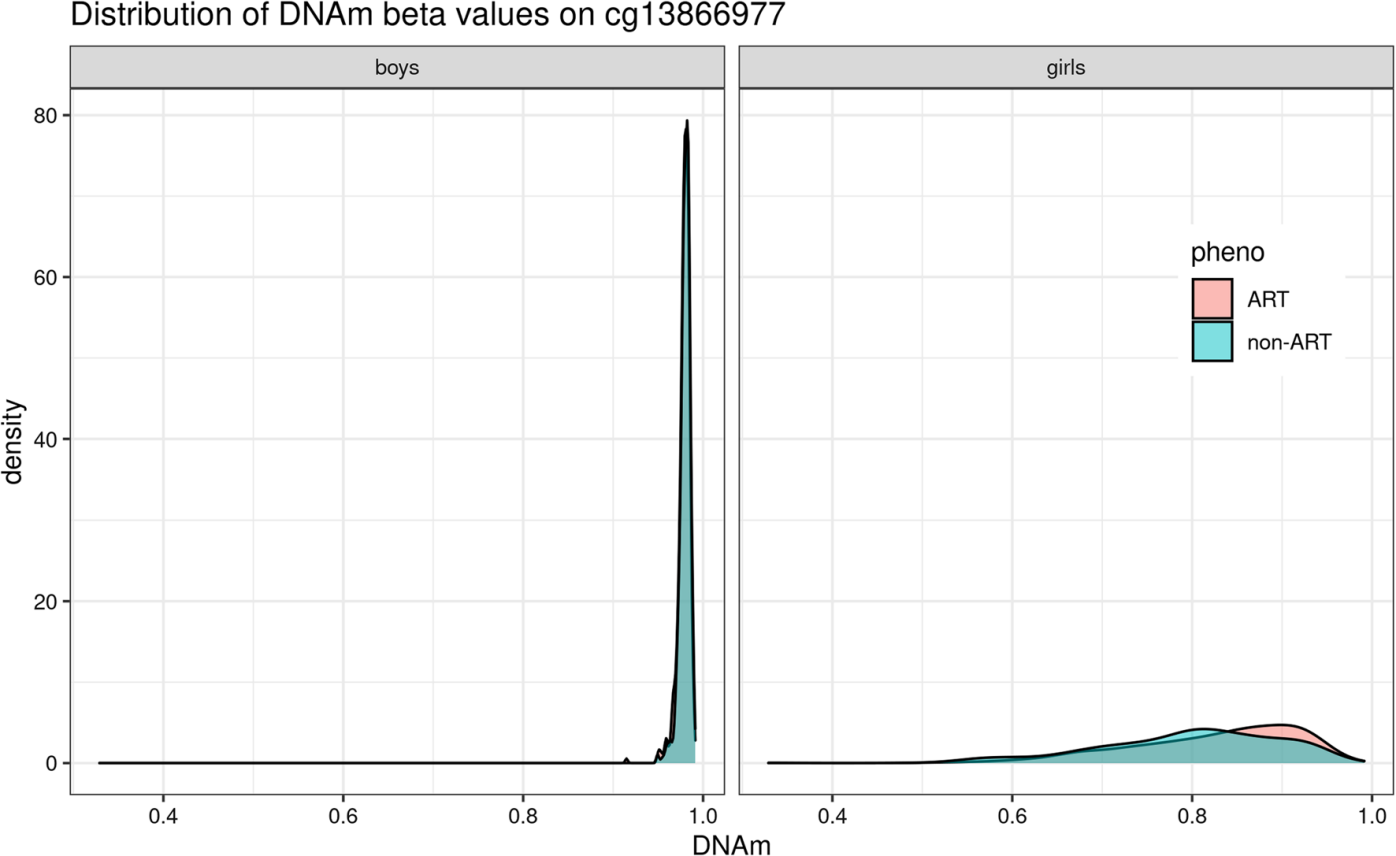
The distribution of methylation $\beta$ values at cg13866977 differs between girls and boys. This CpG is located within a regulatory region and the *AMOT* gene. There is a significant difference in methylation level between girls conceived by ART and girls conceived naturally

Our results also showed that the DNAm level at cg13866977 was close to 1.0 in boys, implying that the cytosine at this site is fully methylated. In girls, it was mostly above 0.7 (Fig. 10). Since DNAm signals mainly reflect the level of transcription, we investigated whether transcription factors (TFs) predicted to bind to cg13866977 preferentially bind to the unmethylated or methylated sequence. The output of the search in JASPAR and MeDReaders indicated that none of the seven TFs bind to the methylated sequence (Additional file 6, also available online in the GitHub repository). This suggests that high methylation at this CpG might signal the inactivation of this regulatory region. Moreover, the methylation state was higher among girls conceived by ART than those conceived naturally (effect size = 0.32). The effect size did not change appreciably when we adjusted for parental DNAm at this site (effect size = 0.33). Again, the independent dataset from the CHART study showed a similar trend of association (Additional file 1: Figure S7), except for two other CpGs, cg05177782 and cg09912589, that were positively correlated with cg13866977 (see Fig. 5). Although these results suggest that the regulatory region within *AMOT* is less active after the ART procedure in girls, the specific function of this activation remains to be elucidated.

### Differential DNAm in boys point to a pseudogene with unknown function

The most significant CpGs in the boys-only analysis, cg00920314 and cg04516011, were both located within *UBE2DNL*. The NCBI gene database (https://www.ncbi.nlm.nih.gov/gene) classifies *UBE2DNL* as a pseudogene, but the ExpressionAtlas database [84] (https://www.ebi.ac.uk/gxa/) reports that it is expressed in testes. According to UniProt, *UBE2DNL* is inactive because it lacks a cytosine in the active site (https://www.uniprot.org/uniprotkb/Q8IWF7). There is mounting evidence pointing to pseudogenes as playing important roles in gene regulation instead of simply being evolutionary relics of inactive genes [85, 86]. This notion has garnered additional support through the application of high-throughput sequencing technologies enabling genome-wide characterizations of pseudogenes [87–89]. Similar to our findings, several of the transcribed pseudogenes identified in a previous study by Zheng et al. [87] were also found to be either transcribed exclusively in testes or were particularly active in those tissues. This pattern of testis-specific pseudogene transcription has also been reported by others [90, 91]. The association with *UBE2DNL* in our data also appears to be credible for three reasons: *1)* the CpGs remained significant even after adjusting for covariates (see Additional file 2), *2)* there is a highly significant DMR in this region, and *3)* the data from the independent CHART cohort showed a DMR in this pseudogene (data not shown) and differential DNAm at these two CpGs in ART vs. non-ART boys (Additional file 1: Figure S8).

### DMRs co-located with genes involved in key developmental processes

The most significant DMR (chrX:118,699,347–118,699,412 in GRCh37) in the girls-only analyses of the MoBa cohort is located within the promoter of three genes (see Fig. 8B and Additional file 1: Table S2 for details). This promoter is active in cord blood, and the genes linked to this promoter are important for immune response, mitochondrial processes, and chromosome segregation. (See the references in Additional file 1: Table S2.) Specifically, one of the genes controlled by this promoter is ‘STING1 ER exit protein 1’ (*STEEP1*, previously called CXorf56, Fig. 8B). Mutations in *STEEP1* cause X-linked intellectual disability and other neurological disorders [92, 93]. The DMR encompassing *STEEP1* was also found to have a lower level of DNAm in ART compared to non-ART newborns, suggesting that the expression of these genes might be up-regulated by ART. The link with *STEEP1* needs to be verified in other similar cohorts when they become available.

Another significant DMR (chrX:152,989,492–152,990,345 in GRCh37) in the girls-only analyses was co-located with a promoter (ensembl ID: ENSR00000249590, see Fig. 8A), which, according to a search in GeneHancer DB, is either a putative promoter or enhancer for nine genes (see Additional file 1: Table S2 for more details). Six of these genes encode proteins that form part of a network, according to the results of text mining and co-expression arrays (STRING DB, https://version-11-5.string-db.org/cgi/network?networkId=bWgRg6ih0nDV). Deletions or duplications in many of these genes have been reported to cause different impairments and diseases [94–100], with autism featuring prominently among these clinical manifestations. Right next to this DMR (chrX:152,989,492-152,990,345 in GRCh37), the boys-only analysis revealed another significant DMR (chrX:153,046,451-153,046,767 in GRCh37) co-located with a promoter (ensembl ID: ENSR00002105690, see Fig. 8A) that is active in cord blood. This promoter regulates four genes that are important in the developmental processes of various tissues, including neurons (Additional file 1: Table S2). These indirect connections with autism and neurodevelopment are particularly noteworthy, given previous reports indicating that neurodevelopmental outcomes differ in children conceived by ART [101, 102], but not always [103, 104].

Lastly, we also checked for any common features among all the genes that co-localized with all the significant DMRs. The STRING protein–protein interaction database (https://version-11-5.string-db.org/cgi/network?networkId=b1fEsljWdfBy) indicates that five of the 13 genes found in all the significant DMRs in the girls-only analysis are involved in the X-linked monogenic disease (DOID:0050735, https://diseases.jensenlab.org/).

### Strengths and weaknesses

A major strength of our study is the large size of the MoBa sample, enabling a more powerful exploration of questions related to ART and infertility. Additionally, the trio design enabled adjusting for parental DNAm in the regression models, which is essential to correct for other DNAm-dependent parental characteristics as a possible reason for the observed associations in the newborns. Another strength is the mandatory reporting of any use of ART to the Norwegian Medical Birth Registry, including the specifics of the ART procedure used to achieve pregnancy. This ensures virtually complete case ascertainment and a detailed assessment of different ART procedures. Combined with the comprehensive data from questionnaires on relevant covariates, the depth of information on these trios is unparalleled. Furthermore, the DNAm data were generated on the more comprehensive Illumina EPIC array, which is a significant technical leap over its predecessors (Illumina’s 27K and 450K Beadchips) in terms of its genomic coverage of regulatory elements, reliability, and reproducibility [105]. One shortcoming of our study is the lack of a well-powered replication cohort with which to compare and validate our findings. EWASs of ART have been far and few between. To our knowledge, the only available dataset was CHART—a small cohort from Australia. Due to differences in the established quality control and analysis pipelines for MoBa and CHART, we were not able to apply the exact same model to both samples. Nevertheless, the model applied to CHART was a close approximation to the main model applied to MoBa. Reassuringly, the results of the main XWAS model in the CHART cohort showed the same trends as observed in the MoBa cohort, despite CHART being significantly smaller and stemming from a different population than MoBa.

## Conclusions

To summarize, our results showed that, for newborns conceived with the help of ART, there were more differentially methylated CpGs and DMRs in girls than boys, with a slightly lower genome-wide methylation in girls and the opposite pattern in boys. Adjustment for several confounders known to be associated with cord-blood DNAm did not affect the associations, nor did adjustment for parental DNAm, which makes it less likely that parental characteristics were responsible for the observed associations in the newborns. Moreover, our downstream bioinformatic analyses revealed that several of the identified genes were expressed in tissues that are relevant for ART and sex. Furthermore, a number of the genes were associated with neurodevelopment and intellectual disability, which is consistent with previous reports of significant differences in neurodevelopment between newborns conceived by ART and those conceived naturally. More generally, our study fills an important knowledge gap in that it provides an easily adaptable analytic pipeline to investigate the contributions of X-linked CpGs to subfertility and other traits. Its application to the reanalysis of previously published EWASs, such as those available in the EWAS Open Platform [106] and the GEO repository, may facilitate the discovery of additional genes and loci that might have been missed by focusing solely on autosomal CpGs.

## Methods

### Discovery cohort—MoBa

MoBa is a large population-based pregnancy cohort study in which pregnant women were recruited across Norway from 1999 through 2008 [63]. Fathers were invited from 2001 onward, which explains the lower number of fathers (75,000) compared to mothers and newborns. The participation rate was 41% among the MoBa mothers. Overall, MoBa includes 114,000 children, 95,000 mothers, and 75,000 fathers. Blood samples were initially drawn from the parents at approximately 18 weeks of gestation, and later from the mother and the umbilical cord after delivery [107]. The current analyses were done on a subset of the MoBa data which was included in the ‘Study of Assisted Reproductive Technology’ (START) project [62]. The current MoBa dataset included 963 trios in which the newborn was conceived using ART and 982 randomly sampled trios in which the newborn was conceived naturally (i.e., by coitus). DNAm in both of these groups of trios was measured using the Illumina Infinium Methylation EPIC BeadChip (Illumina, San Diego, USA) with $\sim$850,000 CpG sites. The inclusion criteria consisted of all of the following: *1)* The child was born in the period 2001–2009, *2)* the child was a singleton newborn with a record in the Norwegian Medical Birth Registry, *3)* the mother filled out and returned the first MoBa questionnaire at around week 17 of gestation, and *4)* blood samples were available for the whole trio (child, mother, and father).

In Norway, fertility clinics are mandated to report any ART conception to the national birth registry. We defined ART as ‘any ART’ (excluding intrauterine insemination) and coded it as a binary variable (ART vs. non-ART). As information on the ART procedure was missing for 79 of the trios, these were excluded from the analysis.

### DNAm measurements in the discovery cohort

DNA samples from the ART and non-ART trios in the MoBa cohort were shipped to Life & Brain GmbH in Bonn, Germany, for further sample processing and measurement of DNAm on the Illumina Infinium Methylation EPIC BeadChip platform (Illumina, San Diego, USA). Extensive details regarding the quality control (QC) pipeline used for data cleaning have been provided in our previous work [62]. Briefly, we established a QC pipeline based on the RnBeads package [108] using the statistical programming language R [109]. Cross-hybridizing probes and probes in which the last three bases overlapped with a SNP were removed from the analyses. Additionally, probes with a detection *p* value above 0.01 were removed. The greedycut algorithm was then applied to remove probes and samples showing outlying DNAm values. This procedure minimizes the false positive rate and maximizes the sensitivity when the retained measurements are considered as prediction for the reliable ones. The remaining DNAm data were corrected for background noise using the enmix.oob function [110].

We extracted DNAm data on the X chromosome only and applied BMIQ [111] to normalize the Type I and Type II probes [112]. We then checked for multimodality of DNAm per CpG for girls and boys separately using the gaphunter function in the minfi R package [113, 114]. Crucially, the QC functions applied to the data did not combine any information across samples, which is essential to keep the analyses separate for males and females due to their distinct modalities. The total number of probes on the X chromosome remaining for the current analyses was 16,841, out of the initial 19,090 X chromosome probes present on the EPIC array.

Figure 1 provides an overview of the analytic pipeline and study population, and the sections below provide additional details.

### Statistical analyses in the discovery cohort

*Regression models.* In preparation for the XWAS of the MoBa cohort, we used the logit2() function from R package minfi [114] to transform $\beta$-values for DNAm into M-values, since M-values are more amenable to statistical tests [115]. Four regression models were fit for boys and girls separately to estimate the difference in methylation levels between the ART and non-ART newborns. This stratification by sex is necessary because of the distinctly different overall DNAm profiles for girls and boys on the X chromosome. In previously published studies, a number of variables were reported to be associated with DNAm in cord blood and with the use of ART, including mother’s age, smoking status, BMI, and whether she was primiparous. These variables were included as potential confounders in the model, i.e., CpG $\sim$ ART + maternal age + maternal smoking + maternal BMI + primiparity (referred to as the ‘main model’; see also Fig. 1). Although all samples were randomly placed on the bisulfite conversion plates before measuring DNAm, the regression model also included plate ID as a random effect to adjust for batch effects. As DNAm levels associated with parental infertility may confound the XWAS results in the newborns, we ran additional models where we adjusted for maternal methylation in the boys-only analysis and for both maternal and paternal methylation in the girls-only analysis (referred to as the ‘adjusted model’). These adjustments were included in the models as fixed effects, separately for each CpG; for more details, please refer to our previous publication [62]. Moreover, we extended each of the two aforementioned models by including further adjustments for gestational age and birthweight (see Fig. 1). Linear mixed models were implemented using the rint.reg function in the R package Rfast [116, 117].

*Controlling for inflation of the test statistics.* Additional file 1: Figure S1 depicts the density curves of the DNAm values ($\beta$-values) in the newborns according to sex and type of probe on the Illumina EPIC array. The methylation patterns are distinctively different in males and females, as has also been reported by other studies (e.g., [59]). Notably, the middle portion of the distribution for females typically exhibits a bump, as a consequence of XCI, whereas males exhibit higher densities at the opposite ends of the distribution. As females have two copies of the X chromosome, and one copy is silenced through XCI, the distribution of the average DNAm is flatter in females than males.

As pointed out by several reports [118–120], large-scale hypothesis testing of high-dimensional data (e.g., those stemming from a GWAS, EWAS, or XWAS) may be prone to heavily inflated type I error when using the theoretical null distribution to assess the significance of the *p* values. We, therefore, used the R package BACON [120] to re-scale the raw z-statistics from the XWAS. BACON is a Bayesian method that controls the false positive rate and accounts for potentially poorly calibrated test statistics while preserving statistical power. We chose BACON over competing methods because it is flexible and can handle a larger proportion of true associations [120, 121]. As can be seen in Additional file 1: Figure S2, BACON reduced inflation substantially in girls but had a negligible effect in boys. After this correction, we applied a false discovery rate (FDR) $< 0.01$ to select CpGs that were significantly associated with ART in our sample.

### Consistency of significant findings in the discovery cohort

We applied a bootstrapping scheme to the XWAS results to evaluate the consistency with which a significant CpG was identified as being significant. We created 1000 bootstrap samples with replacement separately for girls and boys, ensuring an equal proportion of ART and non-ART cases as in the original MoBa dataset. We then reran the analysis using the same main model for each of the bootstrap samples and determined the proportion of times each CpG was found to be significant (using the same significance threshold as previously).

### Co-methylated CpGs and DMR detection in the discovery cohort

We retrieved the annotation tracks from Ensembl BioMart [122] (http://www.ensembl.org) using the R package biomaRt [123, 124] and generated a regional plot of the association results. This regional plot was subsequently combined with a co-methylation (correlation) plot of neighboring CpG sites flanking the significant CpGs. The correlation of DNAm values was calculated and plotted using ggstatsplot [125]. The rationale for this analysis is that if the biological functions of two CpGs are correlated, their DNAm levels are expected to change in the same way between ART and non-ART samples.

We chose the dmrff R package [126] to identify differentially methylated regions (DMRs). This choice was based on the results of a recent paper demonstrating the superior performance of dmrff to four frequently used methods for DMR detection: DMRcate, comb-p, seqlm, and GlobalP [127]. Finally, the R package karyoploteR (part of Bioconductor [128]) was used to visualize genomic features superimposed on a linear representation of the X chromosome [129].

### Downstream bioinformatic analyses in the discovery cohort

The most significant CpGs and DMRs (both at FDR $<0.01$) from the above analyses were subjected to a series of downstream bioinformatic analyses to unravel the biological processes that might be influenced by DNAm at these CpGs. Briefly, we searched the MeDReaders database [130] (http://medreader.org/) and adapted data from Yin et al. [131] to retrieve information about transcription factors (TFs) that preferentially bind to methylated DNA. (The table is available in the GitHub repository.) We also used JASPAR2022 [132] tracks in the ensembl genome browser (http://grch37.ensembl.org/Homo_sapiens/) to check which TFs bind to the significant CpGs detected in our XWASs. GeneHancer [81] was used to find possible targets of promoter and enhancer regions that are co-located with our results. Further, to obtain information on mRNA transcription and protein expression, we searched ExpressionAtlas [133] (https://www.ebi.ac.uk/gxa/home) and HumanProteinAtlas  [134] (https://www.proteinatlas.org/). Finally, information about gene and protein functions and interactions was gathered via GeneCards [135], UniProt [136], and STRING db [137].

### External cohort and analysis

To perform an external check of the findings from the MoBa cohort, we analyzed the DNAm data from the Australian CHART cohort. CHART consists of 547 adults conceived with the use of IVF and 549 naturally conceived controls [64, 65, 138]. In a subsample of 149 ART-conceived and 58 non-ART neonates (see Fig. 1), DNAm was measured in DNA isolated from neonatal blood spots (Guthrie spots) using the Illumina EPIC array. Data were preprocessed using the MissMethyl R package [139], and low-quality and cross-reactive probes were removed from further analysis [15]. Cell composition was estimated using the Bakulski cord-blood cell reference method [140]. Maternal smoking during pregnancy was predicted using a DNA Methylation score [141]. Linear regression modeling was performed using the limma R package [142], with the model: CpG $\sim$ ART + maternal smoking + sample_plate. The analyses were run separately for boys and girls. The preprocessing and analysis pipelines for the CHART and MoBa datasets differ because these were established through separate research processes, and the data were collected differently, were stored in different countries, and could not be accessed by the same researchers.

## Supplementary information

**Additional file 1**. Supplementary tables and figures.

**Additional file 2**. All significant single CpGs.

**Additional file 3**. All significant DMRs.

**Additional file 4**. Bootstrapping results for boys-only analyses.

**Additional file 5**. Bootstrapping results for girls-only analyses.

**Additional file 6**. Transcription factor classification by JASPAR and MeDReaders.

## Data Availability

The MoBa and START data are available from the NIPH, but restrictions apply regarding the availability of these data, which were originally used under specific approvals for the current study and are therefore not publicly available. Data are, however, available from the authors upon reasonable request and after approval by relevant authorities and the NIPH. The CHART data have been deposited in the Gene Expression Omnibus repository, under accession number GSE131433. The scripts for all the above analyses as well as all the results of calculations are available in the GitHub repository at https://github.com/folkehelseinstituttet/X-factor-ART.
