# Supplementary material for: The X-factor in ART: does the use of assisted reproductive technologies influence DNA methylation on the X chromosome?
Source: Hum Genomics. 2023 Apr 21;17:35. doi: 10.1186/s40246-023-00484-6 (PMC10122315; doi:10.1186/s40246-023-00484-6)
Supplement: Supplementary file 1 — Additional file 1. Supplementary tables and figures. [file 40246_2023_484_MOESM1_ESM.pdf]

Additional material

The X-factor in ART: does the use of Assisted Reproductive Technologies influence DNA methylation on the X chromosome?

Julia Romanowska[1,2,*], Haakon E. Nustad[3], Christian M. Page[1,4], William R.P. Denault[1,5], Jon Bohlin[1,6], Yunsung Lee[1], Maria C. Magnus[1], Kristine L. Haftorn[1], Miriam Gjerdevik[1,7], Boris Novakovic[8,9], Richard Saffery[8,9], Håkon K. Gjessing[1,2], Robert Lyle[1,10], Per Magnus[1], Siri E. Håberg[1], and Astanand Jugessur[1,2]

March 21, 2023

Affiliations:
[1] Centre for Fertility and Health, Norwegian Institute of Public Health, 0213 Oslo, Norway
[2] Department of Global Public Health and Primary Care, University of Bergen, 5020 Bergen, Norway
[3] Deepinsight, 0154 Oslo, Norway
[4] Department of Mathematics, Faculty of Mathematics and Natural Sciences, University of Oslo, 0315 Oslo, Norway
[5] Department of Human Genetics, University of Chicago, Chicago, IL 60637, USA
[6] Department of Method Development and Analytics, Norwegian Institute of Public Health, 0213 Oslo, Norway
[7] Department of Computer Science, Electrical Engineering and Mathematical Sciences, Western Norway University of Applied Sciences, 5020 Bergen, Norway
[8] Murdoch Children's Research Institute, Melbourne, Victoria 3052, Australia
[9] Department of Paediatrics, University of Melbourne, Victoria 3010, Australia
[10] Department of Medical Genetics, Oslo University Hospital and University of Oslo, 0424 Oslo, Norway

**Corresponding author:*
Julia Romanowska, PhD
Department of Global Public Health and Primary Care
University of Bergen
5020 Bergen, Norway
E-mail: Julia.Romanowska@uib.no

List of Figures

S1 DNAm overall density 3
S2 QQ plots, all models 4
S3 Volcano plots, all models 5
S4 DMRs location, model 2 6
S5 DMRs location, model 3 7
S6 DMRs location, model 4 8
S7 DNA methylation at two CpGs within the *AMOT* and *EIF2S3* genes in CHART dataset. 9
S8 DNA methylation at three CpGs within the *AMOT* gene in MoBa dataset. . 10
S9 DNA methylation at two CpGs within the *UBE2DNL* gene in CHART dataset. 11
S10 DNA methylation at two CpGs within the *UBE2DNL* gene in MoBa dataset. 12

List of Tables

S1 Genes found to be possibly regulated by regulatory regions that co-localized with the most significant XWAS findings. 13
S2 Genes found possibly regulated by regulatory region colocalized with the most significant differentially methylated regions (DMRs). 14

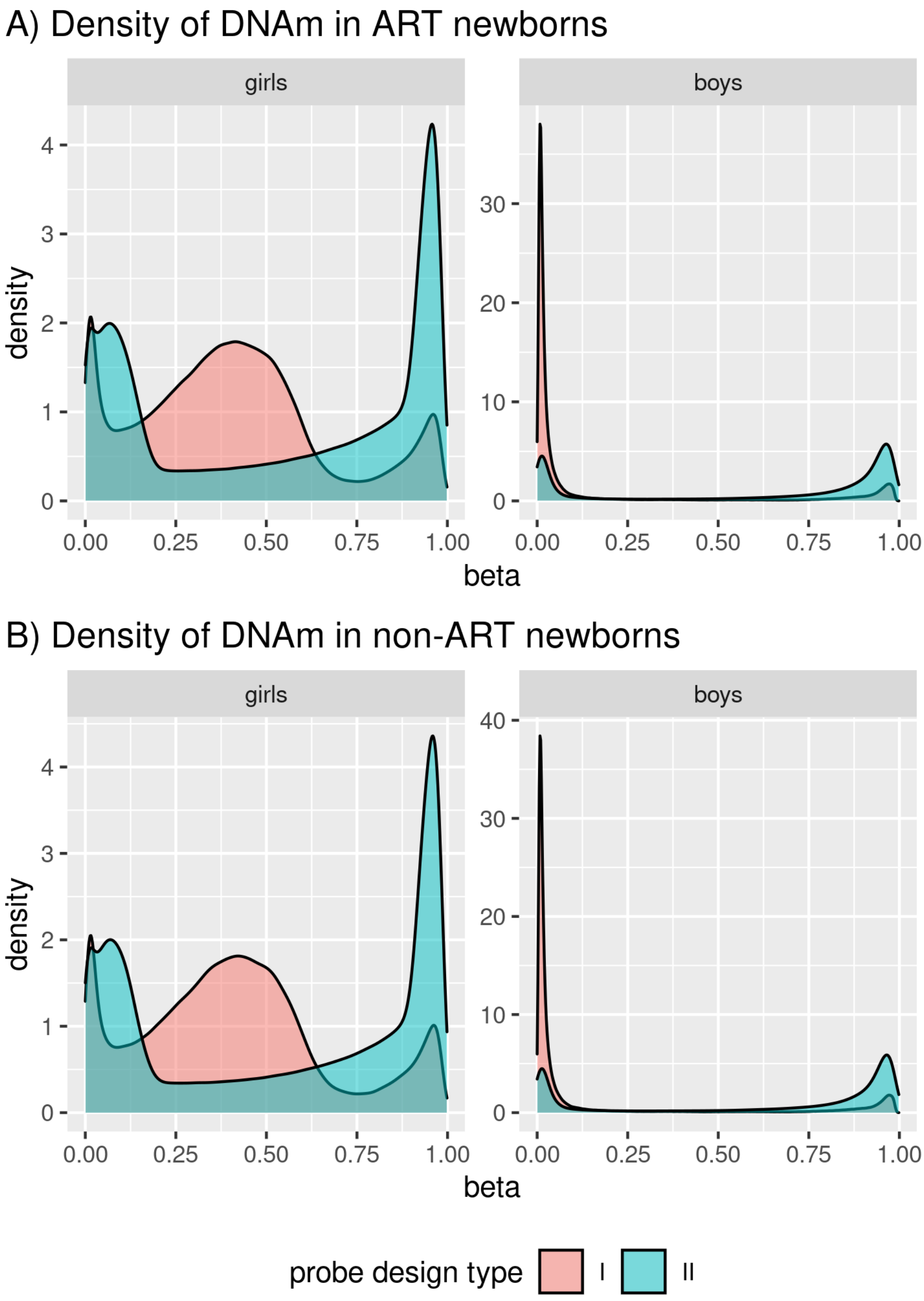

Figure S1: Sex-stratified density plots of the DNAm β-values on X chromosome for ART and non-ART children according to Type I and Type II probes on the Illumina EPIC array.

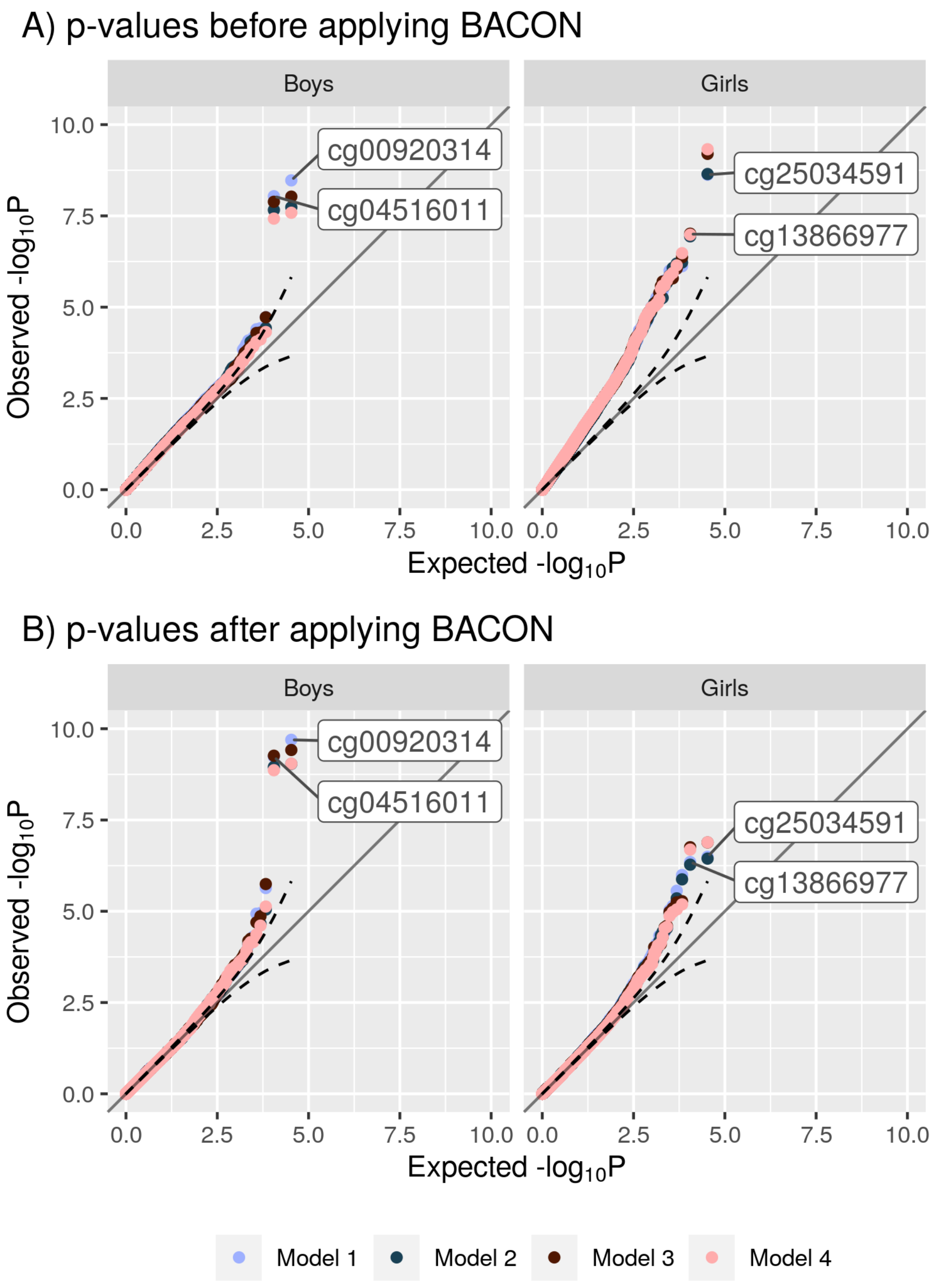

Figure S2: Quantile-quantile (QQ) plot of the observed versus expected $-\log_{10} p$-values of the results for boys and girls before (panel A) and after (panel B) adjustment with BACON.

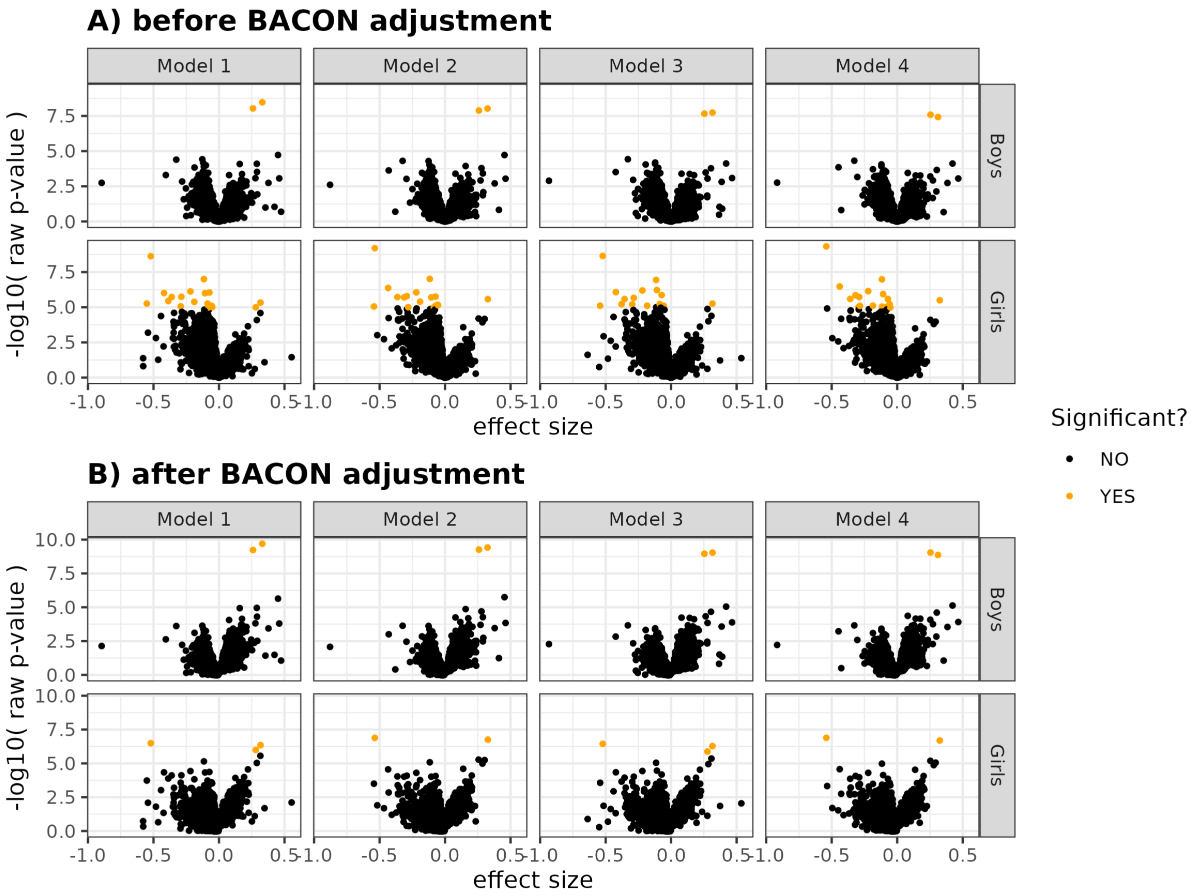

Figure S3: Effect sizes versus $-\log_{10} p$-values for each of the X-linked CpGs included in the analyses. Significant findings at FDR < 0.01 are highlighted in orange.

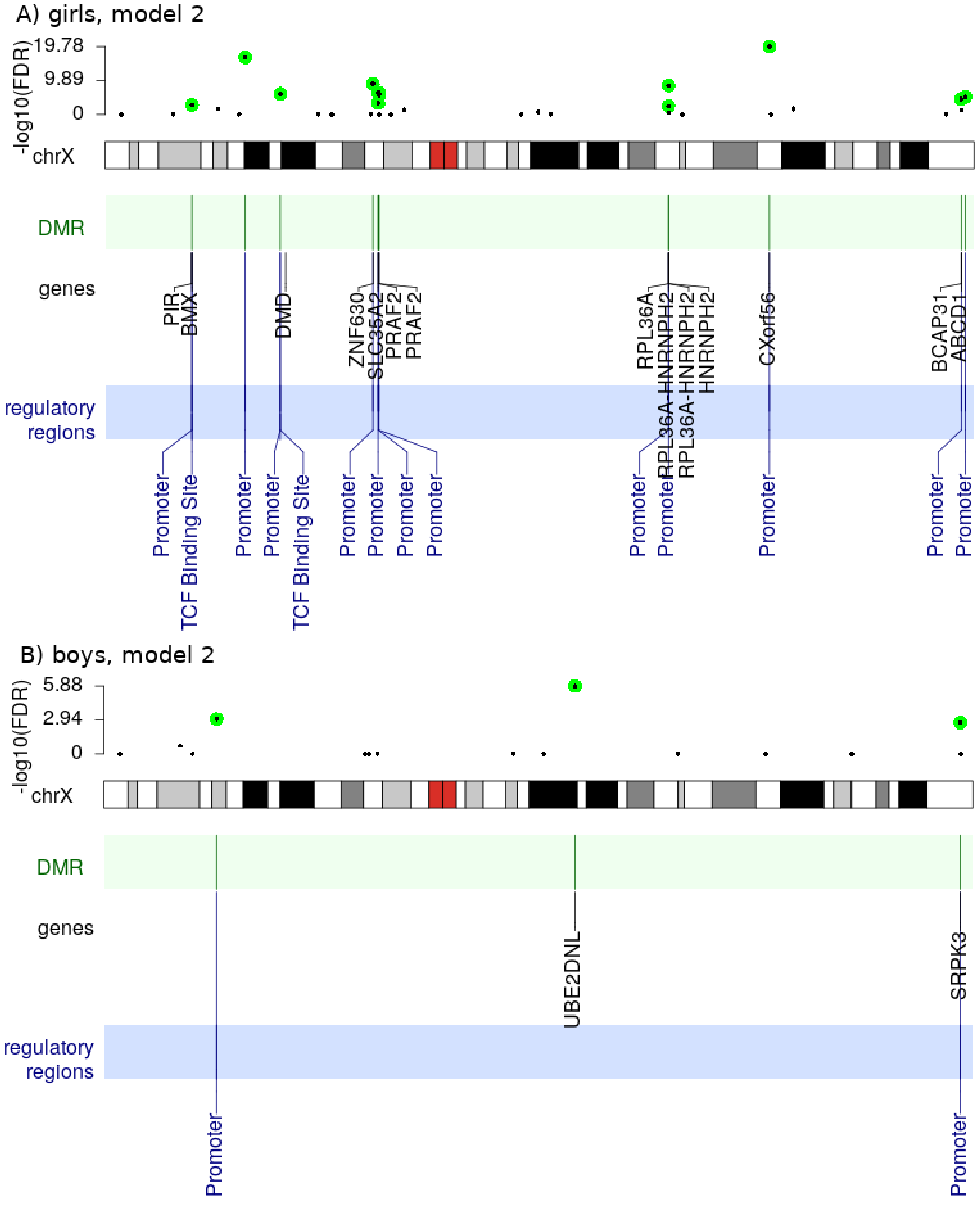

Figure S4: Location of DMRs on the X chromosome for the analyses of Model 2. The top part of each panel shows p-values for all the DMRs that contained at least three CpGs and the FDR-adjusted p-values < 0.01 are marked green. The bottom part of each figure marks genes and regulatory regions harbored by the significant DMRs.

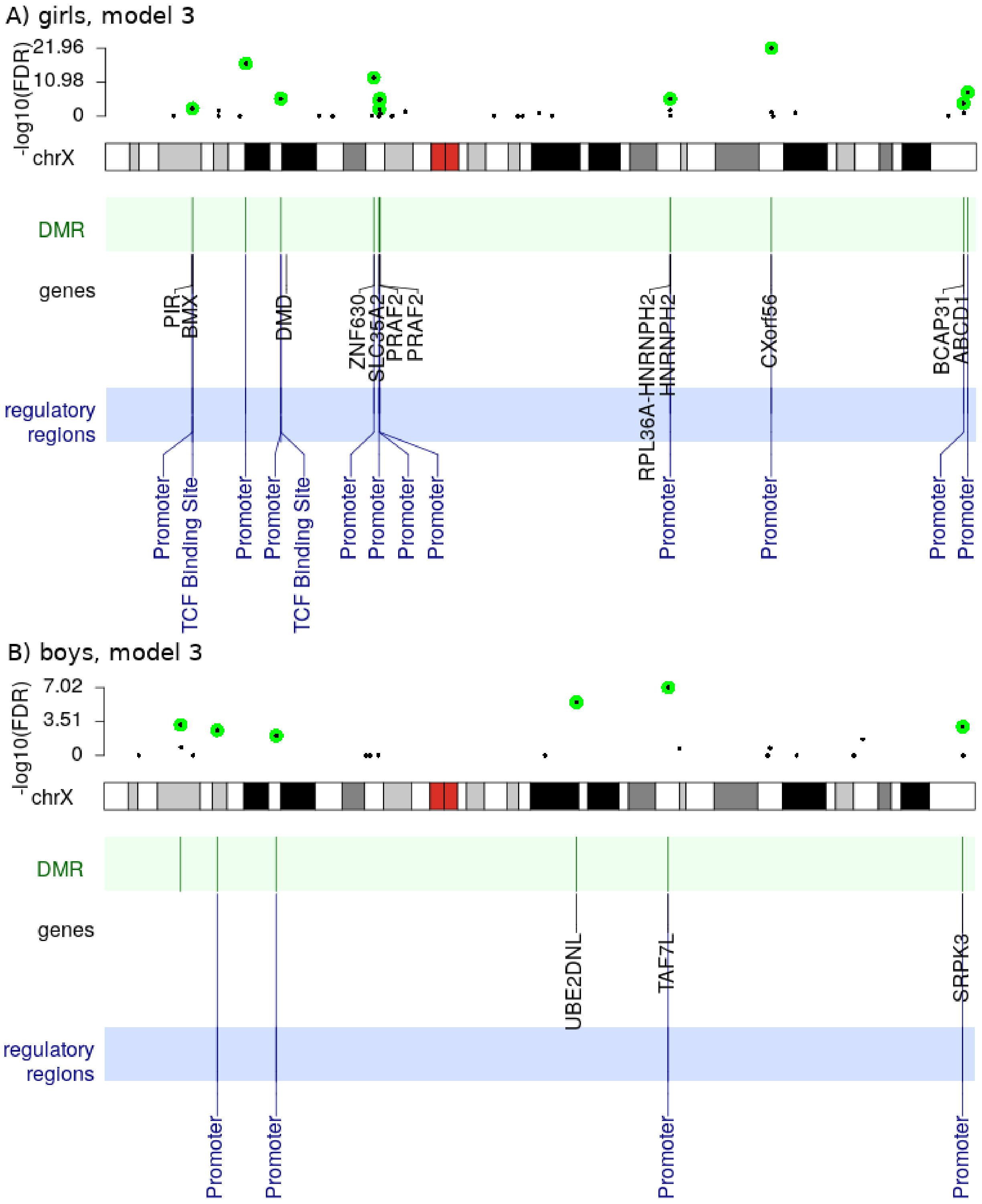

Figure S5: Location of DMRs on the X chromosome for the analyses of Model 3. The top part of each panel shows p-values for all the DMRs that contained at least three CpGs, and the FDR-adjusted p-values < 0.01 are marked green. The bottom part of each figure marks genes and regulatory regions harbored by the significant DMRs.

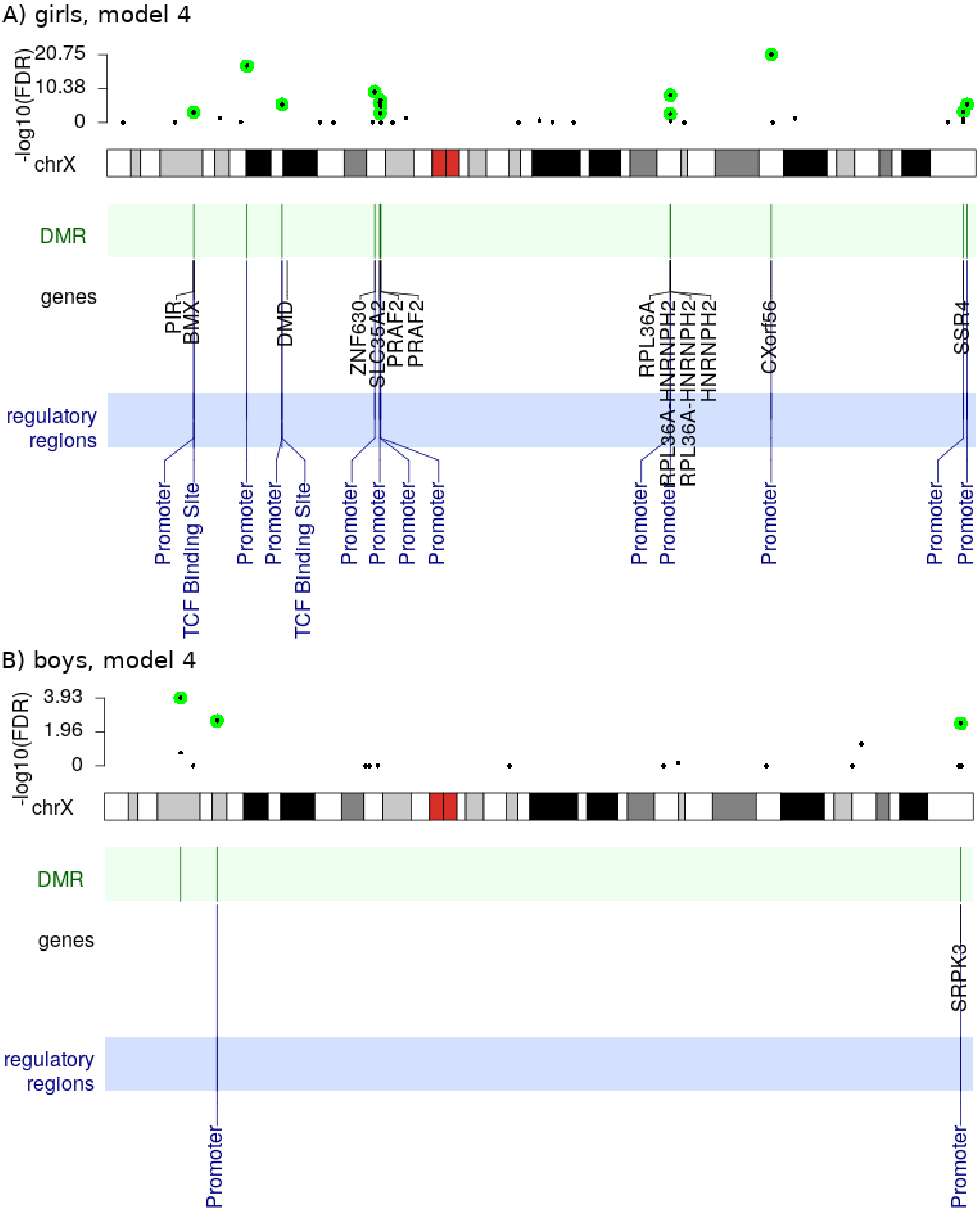

Figure S6: Location of DMRs on the X chromosome for the analyses of Model 4. The top part of each panel shows p-values for all the DMRs that contained at least three CpGs, and the FDR-adjusted p-values < 0.01 are marked green. The bottom part of each figure marks genes and regulatory regions harbored by the significant DMRs.

Figure S7: DNA methylation at two CpGs within the *AMOT* and *EIF2S3* genes in CHART dataset (these two genes were among the significant findings in the girls-only XWAS of the MoBa dataset).
Abbreviations: F = female, M = male, Ctrl = non-ART newborn, ART = newborn conceived through the use of ART.

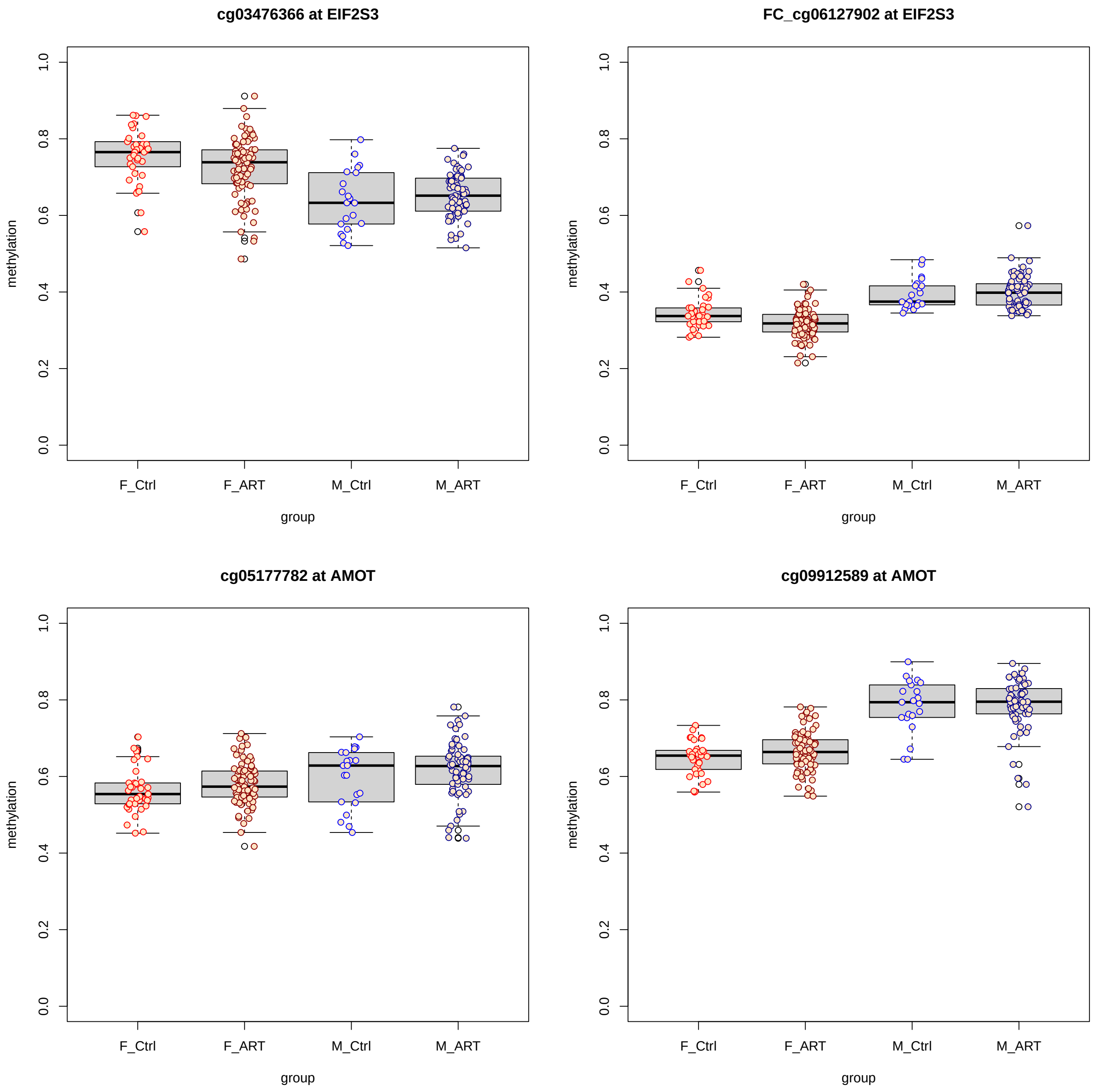

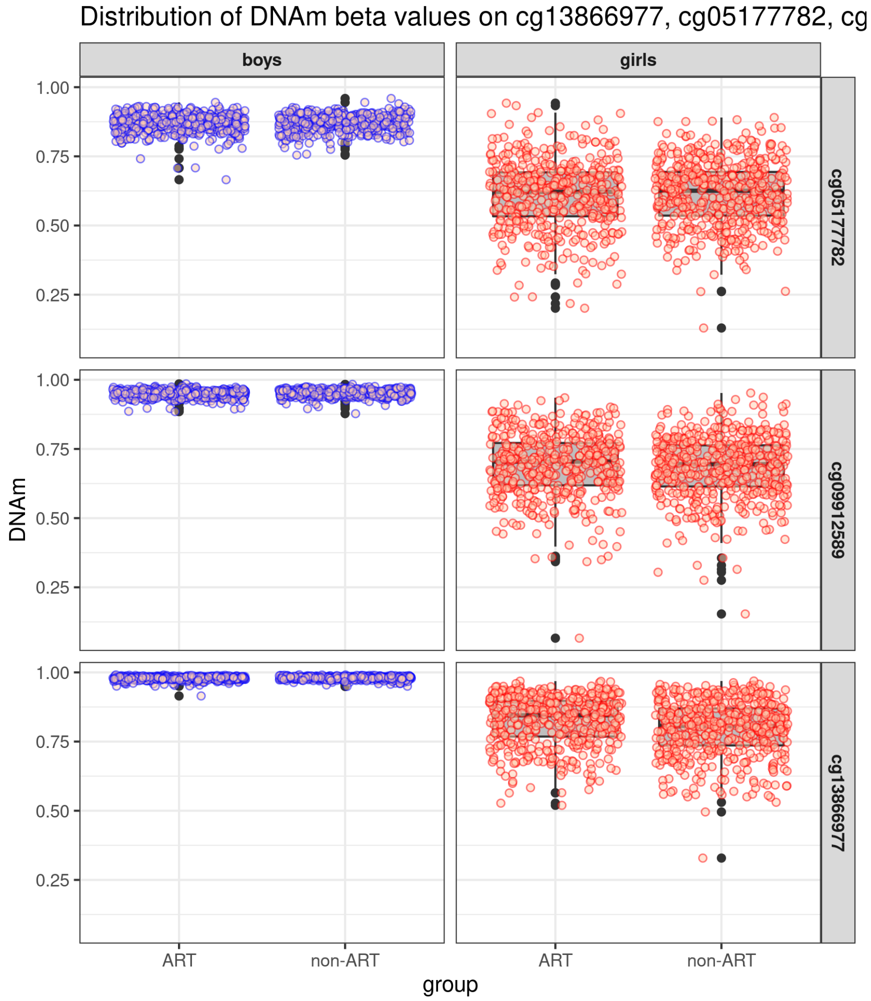

Figure S8: DNA methylation at CpGs within the *AMOT* gene in MoBa dataset — cg05177782 and cg09912589 are shown for comparison with CHART dataset (see Fig. S7), while cg13866977 was found to be significantly associated with ART in MoBa analysis.

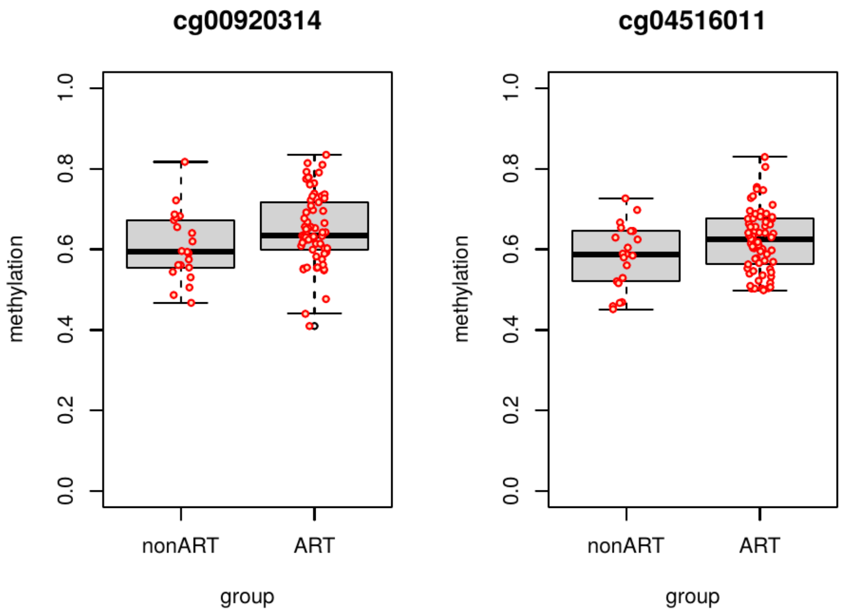

Figure S9: DNA methylation in boys at two CpGs within the *UBE2DNL* pseudogene in the CHART dataset. Note that these two CpGs were significant in the boys-only XWAS of the MoBa data.

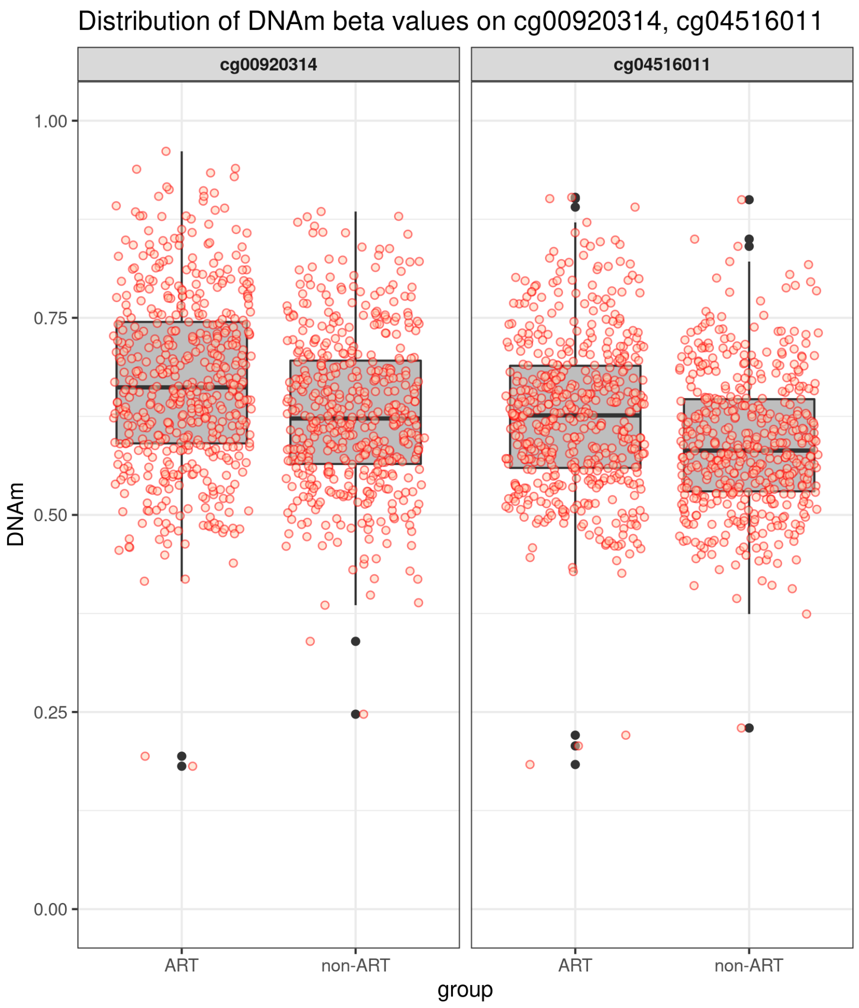

Figure S10: DNA methylation in boys at two CpGs within the *UBE2DNL* pseudogene in the MoBa dataset — for comparison with Fig. S9.

Table S1: Genes found to be possibly regulated by regulatory regions that co-localized with the most significant XWAS findings.

Gene name	Product[(a)]	Function	Expressed in[(b)]	Refs and URLs
		ensembl regulatory ID ENSR00000245352 (co-localized with cg25034591)		
NONHSAG054159.2	lncRNA	Putative role in transcription regulation	Not tissue specific (https://ngdc.cncb.ac.cn/lncbook/gene?geneid=HSALNG0137031)	Statello, L., et al. Nat Rev Mol Cell Biol 22, 96–118 (2021). https://doi.org/10.1038/s41580-020-00315-9
RF00017-7804	SRP_RNA	Putative role in transcription regulation	No data	Akopian, D., et al. Annu Rev Biochem. 2013; 82: 693–721. https://doi.org/10.1146/annurev-biochem-072711-164732
RF00017-7805	SRP_RNA	Putative role in transcription regulation	No data	Akopian, D., et al. Annu Rev Biochem. 2013 ; 82: 693–721. https://doi.org/10.1146/annurev-biochem-072711-164732
HSALNG0137030	lncRNA	Putative role in transcription regulation	Not tissue specific (https://ngdc.cncb.ac.cn/lncbook/gene?geneid=HSALNG0137030)	Statello, L., et al. Nat Rev Mol Cell Biol 22, 96–118 (2021). https://doi.org/10.1038/s41580-020-00315-9
RF00017-7813	SRP_RNA	Putative role in transcription regulation	No data	Akopian, D., et al. Annu Rev Biochem. 2013 ; 82: 693–721. https://doi.org/10.1146/annurev-biochem-072711-164732
EIF2S3	Eukaryotic Translation Initiation Factor 2 Subunit Gamma	translation initiation	Not tissue specific (https://www.proteinatlas.org/ENSG00000130741-EIF2S3/tissue)	https://www.uniprot.org/uniprot/P41091
POLA1	DNA Polymerase Alpha 1, Catalytic Subunit	controls DNA repair and replication	Not tissue specific (https://www.proteinatlas.org/ENSG00000101868-POLA1/tissue)	https://www.uniprot.org/uniprot/P09884
ZFX	Zinc Finger Protein X-Linked	transcriptional activator during processes such as oocyte development and spermatogenesis	Not tissue specific (https://www.proteinatlas.org/ENSG00000005889-ZFX/tissue)	https://www.uniprot.org/uniprot/P17010
KLHL15	Kelch Like Family Member 15	protein ubiquitination	Tissue enhanced (bone marrow) (https://www.proteinatlas.org/ENSG00000174010-KLHL15/tissue)	https://www.uniprot.org/uniprot/Q96M94
RPL9	Ribosomal Protein L9 Pseudogene 7	ribosomal protein, thus involved in translation	Not tissue specific (https://www.proteinatlas.org/ENSG00000163682-RPL9/tissue)	https://www.uniprot.org/uniprot/P32969
		ensembl regulatory ID ENSR00000912938 (a.k.a. ENSR00001768065) (co-localized with cg13866977)		
AMOT	Angiomotin	Important role during formation of new blood vessels in placenta	Tissue enhanced (epididymis/testis, tongue) (https://www.proteinatlas.org/ENSG00000126016-AMOT/tissue)	https://www.uniprot.org/uniprot/Q4VCS5
LHFPL1	LHFPL Tetraspan Subfamily Member 1	Transmembrane protein	Group enriched (brain, salivary gland) (https://www.proteinatlas.org/ENSG00000182508-LHFPL1/tissue)	https://www.uniprot.org/uniprot/Q86WI0
MIR4329	miRNA	Post-transcriptional regulation of gene expression	No data	https://www.nature.com/articles/s41576-020-0263-7 https://www.nature.com/articles/s41576-020-00309-5
piR-39314	piRNA	cleaving tRNA, promoting heterochromatin assembly and methylating DNA	No data	Ozata, D.M., A. et al. Nat Rev Genet 20, 89–108 (2019) https://doi.org/10.1038/s41576-018-0073-3

[(a)] lncRNA = long non-coding RNA; SRP_RNA = signal recognition particle RNA; miRNA = micro RNA; piRNA = Piwi-interacting RNA;
[(b)] n.a. = not applicable

Table S2: Genes found possibly regulated by regulatory region colocalized with the most significant differentially methylated regions (DMRs).

Gene name	Product[(a)]	Function	Expressed in[(b)]	Refs and URLs
		ensembl ID ENSR00000248346 (co-localized with DMR chrX:118,699,347-118,699,412)		
STEEP1 (aka *CXorf56*)	STING1 ER Exit Protein 1	positive regulator of STING signaling, an oligomer essential for proper immune response	No data	https://www.uniprot.org/uniprot/Q9H5V9; Zhang, B., Nature Immunology (2020) https://doi.org/10.1038/s41590-020-0730-5
SLC25A5	Solute Carrier Family 25 Member 5	ADP/ATP translocase 2, important in mitochondrial processes [PMID: 31883789] and was also shown to be part of chromosome segregation process [PMID: 20797633]; "Suppressed expression of this gene has been shown to induce apoptosis and inhibit tumor growth". (via GeneCards)	Not tissue specific (https://www.proteinatlas.org/ENSG00000005022-SLC25A5/tissue)	https://www.uniprot.org/uniprot/P05141; Ito, S., et al. Molecular Cell (2010) https://doi.org/10.1016/j.molcel.2010.07.029; Namba, T., et al., Neuron (2020) https://doi.org/10.1016/j.neuron.2019.11.027
piR-52079-224	piRNA	cleaving tRNA, promoting heterochromatin assembly and methylating DNA	No data	Ozata, D.M., A. et al. Nat Rev Genet 20, 89–108 (2019) https://doi.org/10.1038/s41576-018-0073-3
		ensembl ID ENSR00000249590 (co-localized with DMR chrX:152,989,492-152,990,345)		
SLC6A8	Solute Carrier Family 6 Member 8	"transports creatine into and out of cells. Defects in this gene can result in X-linked creatine deficiency syndrome" (via GeneCards)	RNA expression mainly in mitochondria, low tissue specificity (https://www.proteinatlas.org/ENSG00000130821-SLC6A8/tissue)	https://www.uniprot.org/uniprot/P48029
ABCD1	ATP Binding Cassette Subfamily D Member 1	"plays a role in regulation of VLCFAs and energy metabolism", "Controls also the cellular response to oxidative stress by regulating mitochondrial functions [...]. And finally controls the inflammatory response" (via UniProt)	RNA expression mainly in mitochondria, low tissue specificity (https://www.proteinatlas.org/ENSG00000101986-ABCD1/tissue)	https://www.uniprot.org/uniprot/P33897
BCAP31	B Cell Receptor Associated Protein 31	Chaperone protein in endoplasmic reticulum (ER), important in mitochondrial function	Low tissue specificity; in fibroblasts clustered mainly with other genes of hormone signalling pathway (https://www.proteinatlas.org/ENSG00000185825-BCAP31/tissue)	https://www.uniprot.org/uniprot/P51572
PLXNB3	Plexin B3	"plays a role in axon guidance, invasive growth and cell migration" (via GeneCards)	RNA expression enriched in brain and clustered with other genes of myelination pathway (https://www.proteinatlas.org/ENSG00000198753-PLXNB3/tissue)	https://www.uniprot.org/uniprot/Q9ULL4
PNCK	Pregnancy Up-Regulated Nonubiquitous CaM Kinase	"Phosphorylates and activates CAMK1" (via UniProt)	RNA expression enriched in brain and clustered with other genes of ion transport pathway (https://www.proteinatlas.org/ENSG00000130822-PNCK/tissue)	https://www.uniprot.org/uniprot/Q6P2M8
PDZD4	PDZ Domain Containing 4	Brain-specific protein; ubiquitin protein ligase activity	RNA expression enriched in brain and clustered with other genes of ion transport pathway (https://www.proteinatlas.org/ENSG00000067840-PDZD4/tissue)	https://www.uniprot.org/uniprot/Q76G19
KRT18P48	Keratin 18 Pseudogene 48	pseudogene	n.a.	n.a.
HSALNG0140788	lncRNA	Putative role in transcription regulation	Not tissue specific (https://ngdc.cncb.ac.cn/lncbook/gene?geneid=HSALNG0140785)	n.a.
HSALNG0140785	lncRNA	Putative role in transcription regulation	Not tissue specific (https://ngdc.cncb.ac.cn/lncbook/gene?geneid=HSALNG0140785)	n.a.
		ensembl ID ENSR00002105690 (a.k.a. ENSR00000917836) (co-localized with DMR chrX:153,046,451-153,046,767)		
SSR4	Signal Sequence Receptor Subunit 4	mostly expressed in pancreas (via Human Protein Atlas) and the product is a part of a complex responsible for binding calcium to the endoplasmic reticulum membrane (via UniProt)	Tissue enhanced (pancreas) (https://www.proteinatlas.org/ENSG00000180879-SSR4/tissue)	https://www.uniprot.org/uniprot/P51571; https://www.proteinatlas.org/ENSG00000180879-SSR4/tissue
SRPK3	SRSF Protein Kinase 3	specifically expressed in muscle (via Human Protein Atlas) and the product is required for normal muscle tissue development (via UniProt)	Tissue enhanced (skeletal muscle, tongue) (https://www.proteinatlas.org/ENSG00000184343-SRPK3/tissue)	https://www.uniprot.org/uniprot/Q9UPE1; https://www.proteinatlas.org/ENSG00000184343-SRPK3/tissue
PLXNB3	Plexin B3	expressed specifically in brain (via Human Protein Atlas) and its product, Plexin-B3, is a receptor important in neurogenesis (via UniProt)	Tissue enriched (brain) (https://www.proteinatlas.org/ENSG00000198753-PLXNB3/tissue)	https://www.uniprot.org/uniprot/Q9ULL4; https://www.proteinatlas.org/ENSG00000198753-PLXNB3/tissue
HSALNG0140793	lncRNA	Putative role in transcription regulation	Tissue enhanced (brain, saliva secreting gland) (https://ngdc.cncb.ac.cn/lncbook/gene?geneid=HSALNG0140793)	n.a.

(a) lncRNA = long non-coding RNA; SRP_RNA = signal recognition particle RNA; miRNA = micro RNA; piRNA = Piwi-interacting RNA;
(b) n.a. = not applicable
